# Characterization of brewer's spent grain extracts by tandem mass spectrometry and HPLC‐DAD: Ferulic acid dehydrodimers, phenolamides, and oxylipins

**DOI:** 10.1002/fsn3.3178

**Published:** 2022-12-21

**Authors:** Daniela Becker, Simone Stegmüller, Elke Richling

**Affiliations:** ^1^ Department of Chemistry, Division of Food Chemistry and Toxicology Rheinland‐Pfälzische Technische Universität Kaiserslautern‐Landau Kaiserslautern Germany

**Keywords:** bioactives, Brewer's spent grain, ferulic acid derivatives, hordatines, oxylipins, polyphenols

## Abstract

Brewer's spent grain (BSG) is a major by‐product of the brewing industry which is generated in high amounts. In recent years, sustainable food production has become more and more important. BSG mainly used as cattle feed has gained high interest due to not only its valuable ingredients such as fiber and proteins but also secondary metabolites remaining in BSG after the brewing process and known for many biological effects. In the present study, various methods were applied, such as acetone extraction (A), alkaline hydrolysis followed by ethyl acetate extraction (HE), and acetone extraction of alkaline hydrolysis residue (HA). Compounds present in the respective bioactive extracts were characterized by mass spectrometry to identify the active compounds. Various hydroxycinnamic acid derivatives as well as oxylipins and some dicarboxylic acids, such as azelaic acid, were present in HE and HA extracts. In contrast, some catechins and phenolamides, such as numerous hordatines, as well as oxylipins and phospholipids were detected in A extracts. Quantification using HPLC‐DAD revealed hordatine contents up to 172.2 ± 2.1 μg *p‐*coumaric acid equivalents/mg extract. Hydroxycinnamic acid derivatives content accounted for up to 48% of the total extract (HE extracts) but only around 3% of the total HA extracts. In summary, all extracts contained secondary plant metabolites belonging to different classes, ranging from hydroxycinnamic acids to phenolamides, such as not only hordatines but also oxylipins, which were identified for the first time in BSG.

## Graphical abstract

Different brewer´s spent grain extracts prepared by solid‐liquid extraction were analysed for their main compounds via LC‐MS/MS and HPLC‐DAD. High amounts of hordatines and some other phenolamides as well as oxylipins were detected in these extracts. Extracts prepared by alkaline hydrolysis mainly contained hydroxycinnamic acid derivatives such as ferulic and *p*‐coumaric acid as well as ferulic acid dehydrodimers and also oxylipins.

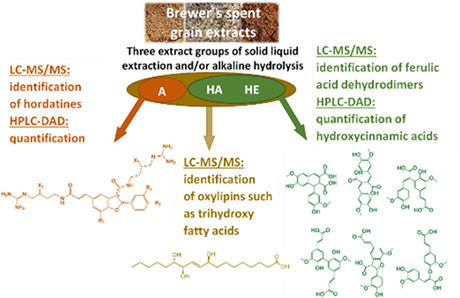

## 1 INTRODUCTION

Beer is the most popular alcoholic beverage worldwide and its annual global production in 2020 accounted for around 1.8 billion hectoliters (Barth, 2021). Its major by‐product is the brewer's spent grain (BSG) with around 14–20 kg/100 L beer (Kunze, 2010; Olajire, 2020) leading to high amounts (up to 39 million tons) of BSG worldwide. As a sustainable and responsible food production has become increasingly important, alternative usages of agri‐food by‐products such as BSG became crucial in recent years. BSG mainly consists of the insoluble part of the malt, which remains after the brewing process. Mainly barley grains and to a lesser extent other cereals, such as wheat or maize, are used (Lynch et al., 2016; Mussatto, 2014). Despite its high nutritional value due to its high protein (19–30% w/w) and fiber (30–50% w/w) content, BSG is often used as cattle feed. In recent years, other applications have been investigated, e.g., as an ingredient for human food to enhance its nutritional value (Merten et al., 2022; Sahin et al., 2021), used in biogas production (Szaja et al., 2020), or as an adsorbent to remove metals from waste water (Lu & Gibb, 2008). It is well‐known that beer is rich in polyphenols such as hydroxycinnamic acids, flavonoids, and also phenolamides, as reviewed by Wannenmacher et al. (2018), but many of these bioactive compounds have been reported to be present in BSG as well (Bonifácio‐Lopes et al., 2020; Verni et al., 2020).

BSG is a lignocellulosic material with cellulose, hemicellulose, and lignin being the most abundant components. Hemicelluloses may be present at up to 40% of the total dry weight (dw) of BSG (Mandalari et al., 2005), and due to cross‐linkers built from ferulic acid dehydrodimers (DiFA), BSG is an interesting source of bound polyphenols (Stefanello et al., 2018). Lignin, a polyphenolic macromolecule, is found at up to 28% BSG dw and consists of not only monomers sinapyl/coniferyl and *p*‐coumaroyl alcohol but also high amounts of polyphenols, such as ferulic acid (FA) or vanillic acid, as part of the lignin structure (Aura et al., 2013; Mussatto et al., 2010). Besides the lignocellulosic compounds in BSG, high amounts of proteins (14%–31%) (Santos et al., 2003; Xiros et al., 2008) have been reported with essential amino acids, such as methionine, tryptophane, or lysine, representing around 30% of the total protein content (Lynch et al., 2016). Moreover, up to 13% of lipids have been found in BSG (Xiros et al., 2008). Niemi et al. reported that the lipids comprised mostly triglycerides (55%), and also free fatty acids (30%), phospholipids (9.1%), and diglycerides (5.7%) (Niemi et al., 2012). Del Río et al. reported similar distributions within lipid extracts of BSG, with palmitic, oleic, and linoleic acids being the predominant free fatty acids (Del Río et al., 2013). Previously, Anness and Reud reported that most lipids from malt remained in BSG but around 30% of lipids were oxidized during mashing (Anness & Reud, 1985), which might lead to modified lipid classes in BSG. Arts et al. reported the presence of hydroxylated fatty acids resulting from enzymatic oxidation during the mashing process (Arts et al., 2007). In line with these findings, trihydroxylated fatty acids have been detected in beer (Esterbauer & Schauenstein, 1977). They belonged to the class of oxylipins, comprising oxidation products of polyunsaturated fatty acids with oxygen as well as their primary oxidation products (Arts et al., 2007).

Regarding the polyphenols in BSG, a distinction between bound and free compounds can be made and has been investigated in different studies (Birsan et al., 2019; Verni et al., 2020). The first step in the extraction process is usually SLE with organic solvents (free polyphenols), followed by alkaline hydrolysis (bound polyphenols), which disrupts ester bonds in the cell walls, leading to the liberation of hydroxycinnamic acids. Free polyphenols mainly consisted of flavonoids like catechin, whereby bound polyphenols such as hydroxycinnamic acid derivatives, particularly FA and *p‐*coumaric acid (*p*CA) were present in 50‐ to 100‐fold higher amounts (Birsan et al., 2019; Verni et al., 2020). Ferulic acid oligomers are also part of the bound polyphenols and were reported to be contained in BSG (Birsan et al., 2019; Moreira et al., 2013; Verni et al., 2020).

We recently investigated the influence on glucose‐metabolizing enzymes with BSG extracts prepared by solid–liquid extraction (SLE) with 60% acetone or alkaline hydrolysis followed by ethyl acetate as well as acetone extraction of alkaline hydrolysis residue (Becker et al., 2021). All of the extraction methods are well‐known procedures and were already used for the extraction of polyphenols (Hernanz et al., 2001; Jay et al., 2008; Meneses et al., 2013). Our approach was to generate different extract groups with varying compounds including solid–liquid extracts containing flavonoids and other polyphenols and extracts from alkaline hydrolysis mainly containing hydroxycinnamic acids. Extensive purification by ethyl acetate extraction and SPE was performed to ensure the elimination of interfering carbohydrates (Aarabi et al., 2016; Michalkiewicz et al., 2008). The respective extracts were characterized by the total phenolic content (TPC) and total flavonoid content (TFC), but individual compounds were not identified yet. In the present study, the same BSG extracts were analyzed by tandem mass spectrometry, and specific compounds were quantified by HPLC‐DAD in a semiquantitative approach in order to identify active compounds.

## 2 MATERIALS AND METHODS

### 2.1 Chemicals

Chemicals were of analytical grade and obtained from Sigma‐Aldrich (Taufkirchen, Germany) unless otherwise stated. HPLC LC–MS grade and supergradient HPLC grade acetonitrile and HPLC LC–MS grade methanol were obtained from VWR Chemicals (Darmstadt, Germany). Formic acid was purchased from Carl Roth (Karlsruhe, Germany), and formic acid Optima LC–MS grade from Fisher Chemicals (Waltham, USA). *Trans*‐ferulic acid (purity 99%), caffeic acid (purity ≥98%), *p*‐coumaric acid (purity ≥98%), *trans‐*cinnamic acid (purity 99%), l‐tryptophan (purity ≥98%), and 3,4‐dimethoxycinnamic acid (purity 99%) were obtained from Sigma‐Aldrich (Taufkirchen, Germany). Nonanedioic acid (azelaic acid) (purity ≥98%), octanedioic acid (suberic acid) (purity ≥98%), (9, 10, 13)‐trihydroxy octadecenoic acid (purity >99%), and (9,12,13)‐trihydroxy octadecenoic acid (purity >99%) were purchased from Larodan (Solna, Sweden).

### 2.2 BSG samples and extracts

Three different batches of BSG were used: one from the conventional Orval Brewery in Belgium (Florenville, Belgium, BSG 3) and two supplied by the brewing group of the Chair of Bioprocess Engineering at the Technische Universität Kaiserslautern (Kaiserslautern, Germany; BSG 1–2). The malts used for these batches are listed in Table 1.

**TABLE 1:** Raw material for extraction and malt composition of BSG as well as nomenclature of BSG extracts (purified by SPE and lyophilized)

Raw material used	Malt used for brewing	Process 1		Process 2		Process 3
		Acetone extraction	Alkaline hydrolysis + ethyl acetate extraction	Alkaline hydrolysis + ethyl acetate extraction	Acetone extraction of hydrolysis residue	Acetone extraction
		Purification by solid‐phase extraction				
BSG 1	Wheat malt (54.3%) and Pilsen malt (45.7%)	A1	HE1			
BSG 2	Weyermann® Vienna malt (100%)	A2	HE2	HE4	HA1	A4
BSG 2 defatted				HE6	HA3	A5
BSG 3	Pilsen malt (90%), caramel malt (9%), peeled, and roasted barley (1%)	A3	HE3	HE5	HA2	A6
BSG 3 defatted				HE6	HA3	A7

Extracts were prepared using three different extraction processes: combinations of (a) ultrasound‐assisted alkaline hydrolysis with 4 M sodium hydroxide (NaOH) followed by ethyl acetate extraction (HE extracts), (b) SLE with 60% acetone (acetone/water: 60/40; v/v; A extracts), and (c) acetone extraction of alkaline hydrolysis residue (HA extracts). All extractions were followed by a purification step using solid‐phase extraction with C18ec material and a lyophilization process as previously described in more detail (Becker et al., 2021). As a result, three different extract groups, labeled A1–A7 (solid–liquid extraction with 60% acetone), HE1–HE6 (alkaline hydrolysis followed by ethyl acetate extraction), and HA1–HA3 (acetone extraction of hydrolysis residue), were obtained (Table 1).

### 2.3 HPLC–MS/MS characterization of BSG extracts

Analysis of the extracts listed in Section 2.2 was performed with HPLC–MS (Appendix S1: Supplement Material A). HPLC conditions were as follows: Synergi 4 μm polar RP 80 Å column (250 mm × 4.6 mm, Phenomenex, Torrance, California, USA); solvent system: A 0.1% formic acid and B acetonitrile; gradient profile: isocratic 10% B for 9 min, from 10 to 25% B over 1 min, isocratic 25% B for 9 min, from 25 to 50% B over 15 min, from 50 to 98% B over 6 min, isocratic 98% B for 10 min, from 98 to 10% B over 0.1 min, and isocratic 10% B for 10 min; flow rate 800 μl/min; injection volume 20–50 μl (depending on HPLC‐MS system used); column oven temperature 30 °C; samples measured at concentrations of 100–1000 μg/ml for extracts and 1–100 μg/ml for reference (depending on HPLC‐MS system used); substances in methanol/formic acid (99.9/0.1; v/v), membrane filtered (0.45 μm) prior injection; and UV detection: 280, 300, and 320 nm. ESI_pos_‐MS(/MS) and ESI_neg_‐MS(/MS) conditions can be found in the supplement materials (Appendix S1: Supplement Material A). Initially, precursor ions were identified from a full scan followed by a product ion scan.

### 2.4 Identification of phenolamides

Identification of phenolamides was achieved by HPLC‐MS as previously described (Becker et al., 2022). Different extract concentrations were measured due to varying phenolamide content, and therefore huge differences in signal intensity to ensure clear identification are as follows: A1: 50 μg/ml, A2: 35 μg/ml, A3: 40 μg/ml, A5 and A6: 25 μg/ml, and A7: 12.5 μg/ml in methanol/formic acid (99.9/0.1; v/v), membrane filtered (0.45 μm) prior injection; and injection volume was set to 20 μl.

### 2.5 Structural elucidation of ferulic acid dehydrodimers and identification of oxylipins

Structural elucidation of DiFAs as well as identification of trihydroxy oxylipins were performed by HPLC‐MS (Appendix S1: Supplement Material A) and product ions scans. HPLC conditions were as follows: Synergi 4 μm polar RP 80 Å column (250 mm × 4.6 mm, Phenomenex, Torrance, California, USA); solvent system: A 0.1% formic acid and B acetonitrile; gradient profile: isocratic 25.1% B for 11 min, from 25.1 to 34.5% B over 30 min, from 34.5 to 98% B over 0.1 min, isocratic 98% B for 7 min, from 98 to 25.1% B over 0.1 min, and isocratic 25.1% B for 12 min; flow rate: 800 μl/min; injection volume: 5–20 μl (depending on the extract solution concentration and expected concentration of the compound) membrane filtered (0.45 μm) prior injection; column oven temperature: 35 °C; and sample concentration in 0.1% formic acid in methanol: 250 μg/ml. ESI_neg_‐MS(MS) conditions can be found in the supplement material (Appendix S1: Supplement Material A).

### 2.6 Quantification of total hordatine content by HPLC‐DAD

Total hordatine content was determined as *p*‐coumaric acid equivalents (*p*CA‐Eq) based as previously described (Becker et al., 2022) using a correction factor (CF) due to the different UV absorption of hordatines and *p*CA which was adapted from published data (Pihlava et al., 2016).

Two independent extract solutions were prepared and each was injected three times (except A2, six injections of the same extract solution). LOD was determined as 0.3 μg/ml and LOQ as 1.0 μg/ml. The precision of the method was determined by inter‐ and intra‐day repetition experiments; and the coefficient of variation was 0.5% for intra‐day and 0.8% for inter‐day experiments. Matrix effects were evaluated by comparison of internal standards response in extract matrix compared to solvent and were determined with a deviation of 5.8%, which was significant (*p* < .05) indicating only slight matrix effects. Detailed information on the method validation can be found in Section 2.9.

### 2.7 Quantification of hydroxycinnamic acids by HPLC‐DAD

Three hydroxycinnamic acids, namely *p*CA, *trans‐*FA, and caffeic acid (CA), were quantified by HPLC‐DAD. Based on the peak area of compounds in relation to the peak area of an IS, an external calibration curve ranging from 2.5–75 μg/ml for *p*CA, 2.5–150 μg/ml for FA, 2.5–25 μg/ml for CA, and 20 μg/ml *trans‐*cinnamic acid as IS was used. Additionally, DiFAs were quantified as FA‐Eq since no pure substances were available. All sample dilutions were prepared in methanol/formic acid (99.9/0.1; v/v). Quantification was performed using HPLC‐DAD (Appendix S1: Supplement Material A).

HPLC conditions were as follows: Synergi 4 μm polar RP 80 Å column (250 mm × 4.6 mm, Phenomenex, Torrance, California, USA); solvent system: A 0.1% formic acid and B acetonitrile; gradient profile: from 15 to 25.1% B over 7 min, isocratic 25.1% B for 4 min, from 25.1 to 34.5% B over 30 min, from 34.5 to 98% B within 0.1 min, isocratic 98% B for 8 min, from 98 to 15% B within 0.1 min, and re‐equilibration for 12 min; flow rate: 800 μl/min; injection volume: 20 μl, membrane filtered (0.45 μm) prior injection; column oven: 35°C; detection wavelength: 300 nm (reference wavelength 440 nm); and extracts were measured at concentrations of 250–1000 μg/ml prepared in methanol/formic acid (99.9/0.1; v/v).

Two independent extract solutions were prepared and each was injected two or three times. LOD was determined for each as 0.7 μg/ml and LOQ as 2.4 μg/ml for *p*CA and CA, and 2.6 μg/ml for FA. The precision of the method was determined by inter‐ and intra‐day repetition experiments; the coefficients of variation were 1.4% (*p*CA), 1.2% (FA), and 2.2% (CA) for intra‐day, and 3.2% (*p*CA), 2.5% (FA), and 2.4% (CA) for inter‐day experiments. Matrix effects were evaluated by indirect regression using spiking method. Within both extract groups (HA and HE) no significant matrix effect was observed for the three investigated hydroxycinnamic acids. Detailed information on the method validation can be found in Section 2.9. Results were multiplied with a substance‐specific CF, which was obtained from the slope of a regression line obtained by plotting concentration (*p*CA, FA, and CA) × area (IS) on the *y*‐axis vs. concentration (IS) × area (*p*CA; FA; CA) on the *x*‐axis. Experiments were performed twice, each comprising three injections. Specific CFs were calculated as follows: *p*CA 0.873; FA 1.301; and CA 1.211. Due to the lack of availability of pure substances, no CFs for DiFAs could be calculated.

### 2.8 Statistical analysis

Results for quantification were presented as means and SD of two independent experiments, each analyzed in duplicate/triplicate. Statistical analyses were performed with Origin 2019G (OriginLab, Northampton, USA) and Excel Office Professional Plus 2016 (Microsoft, Redmond, USA). Data were checked for normality (Anderson Darling test) and homogeneity of variance (Fisher test). Significant differences between and within extraction groups were evaluated using a one‐sample *t*‐test (one sided) (Couallier, 2013; Fahrmeir et al., 2016). Differences were considered significant at *p* < .05; *p* < .01; and *p* < .001 levels.

Matrix effects within quantification methods were evaluated using a two‐sided *t*‐test and checked for (a) differences between internal standard response in extract matrix solution compared to solvent solution (for hordatine quantification) or (b) significant differences between experimental determined *b*‐value, an indicator for multiplicative systemic errors achieved by indirect regression (see Section 2.9 for hydroxycinnamic acids quantification) and theoretical *b*‐value of 0.

### 2.9 Method validation

Calibration curves were checked for normality (David test) and outliers (Nalimov test). The working range of standard substances as well as the IS was examined for linearity. The method sensitivity was assessed by determination of LOD and LOQ of each compound by a calibration approach. Five concentrations (hydroxycinnamic acid quantification: 0.25–5.0 μg/ml for *p*CA; 0.25–2.5 μg/ml for CA and FA; and hordatine quantification: 0.25–2.5 μg/ml for *p*CA) including a blank were prepared in duplicate and analyzed. LOD was calculated as follows:

$$x_{\left(L\;0\;D\right)}=3.8*\frac{s_{y,x}}{b}*\sqrt{1.1+\frac{{\left(\bar{x}\right)}^{2}}{\sum_{i=1}^{n}{\left(x_{i}-\bar{x}\right)}^{2}}}$$

where *x*_LOD_ is the LOD, *S*_*y*,*x*_ is the SD of residuals, *b* is the slope of the calibration curve, $\bar{x}$ is the mean calibration level, and *x*_*i*_ is the content value of the analyte at calibration level *i*. LOQ was calculated as 3.3 times LOD (European Commission; Joint Research Centre, 2016). Precision was evaluated by an inter‐ and intra‐day repetition method. Intra‐day repeatability was assessed by five replicate analyses of one standard concentration; and inter‐day reproducibility was obtained by analysis of one concentration level of the analyte for 5 days in a row. Mean and SD of each experiment were determined, and the coefficient of variance was calculated.

Matrix effects were evaluated by two methods. For hordatine quantification, a comparison of internal standard (20 μg/ml 3,4‐dimethoxycinnamic acid) response in extract matrix and solvent matrix (0.1% formic acid in methanol) was performed. Mean values of all measurements (*n* = 2; triplicates; each replicate: AUC mean value of seven extracts and six *p*CA calibration solutions) were analyzed with regard to their deviation (%). Matrix effects within hydroxycinnamic acids’ quantification was evaluated by indirect regression method using spiking (Wellmitz & Gluschke, 2004). Three different concentrations of FA, *p*CA, and CA (for HE extract) were each added to one HA extract (1000 μg/ml) and one HE extract (250 μg/ml) as follows: for HA extract *p*CA: 5, 7.5, and 20 μg/ml; FA: 10, 17.5, and 40 μg/ml; for HE extract: *p*CA: 20, 25, and 35 μg/ml; FA: 50, 75, and 100 μg/ml; and CA: 2.5, 5, and 7.5 μg/ml. Each extract was analyzed spiked as well as unspiked and each reference spiking solution was also analyzed in pure solvent. Criteria for matrix effects is the *b*‐value which was calculated as follows:

$$b=\frac{x_{3}-x_{1}}{x_{+}}$$

where *x*_3_ = AUC of spiked extract, *x*_1_ = AUC of unspiked extract, and *x*_+_ = AUC of reference spiking solution. Mean *b*‐values were calculated (*n* = 2; duplicates; each replicate includes three spiking levels of each reference substance) and analyzed for significant differences to theoretical *b*‐value of 0.

## 3 RESULTS AND DISCUSSION

BSG extracts were prepared by three different extraction processes as previously described (see Section 2.2, Becker et al., 2021), resulting in three extract groups (HA, HE, and A; Table 1). All methods applied have previously been used to extract polyphenols from BSG. Meneses et al. found 60% acetone to be the most effective solvent among various aqueous, organic, solvent mixtures regarding the total phenolic content of the generated BSG extracts (Meneses et al., 2013). Thus, it was used to generate A1–A7 extracts. Besides enzymatic treatment, alkaline hydrolysis represents the method of choice for the extraction of hydroxycinnamic acids from plant material, whereby concentrations ranging from 1 to 4 M NaOH are normally used (Hernanz et al., 2001; Jay et al., 2008). Ester‐linked hydroxycinnamic acids and also sugars can be released by NaOH and are therefore found in the aqueous extracts from alkaline hydrolysis. Hence, ethyl acetate extraction was used to eliminate liberated carbohydrates from the raw extracts leading to HE1–HE6 extracts. Furthermore, a second extraction step with 60% acetone was performed using the solid residue of the hydrolysis to ensure that all hydroxycinnamic acids were extracted and HA1–HA3 extracts were generated. Additionally, all three extract groups were purified and secondary metabolites were concentrated using SPE with C18ec material (Michalkiewicz et al., 2008). HPLC‐MS/MS analysis showed that the extracts strongly differed regarding their main compounds: A1–A7 extracts contained some polyphenols but were mainly rich in hordatines. HE1‐HE6 extracts were rich in not only hydroxycinnamic acids, which were released from cell wall arabinoxylans by alkaline treatment, as described in the literature (Stefanello et al., 2018), but also trihydroxy oxylipins were detected. HA1–HA3 extracts were generated to ensure almost complete extraction of hydroxycinnamic acids. In general, similar compounds as in HE extracts were identified in HA extracts, namely hydroxycinnamic acids and trihydroxy oxylipins, although the amounts were much lower.

### 3.1 Compound identification in extracts by HPLC‐MS/MS

**FIGURE 1:**
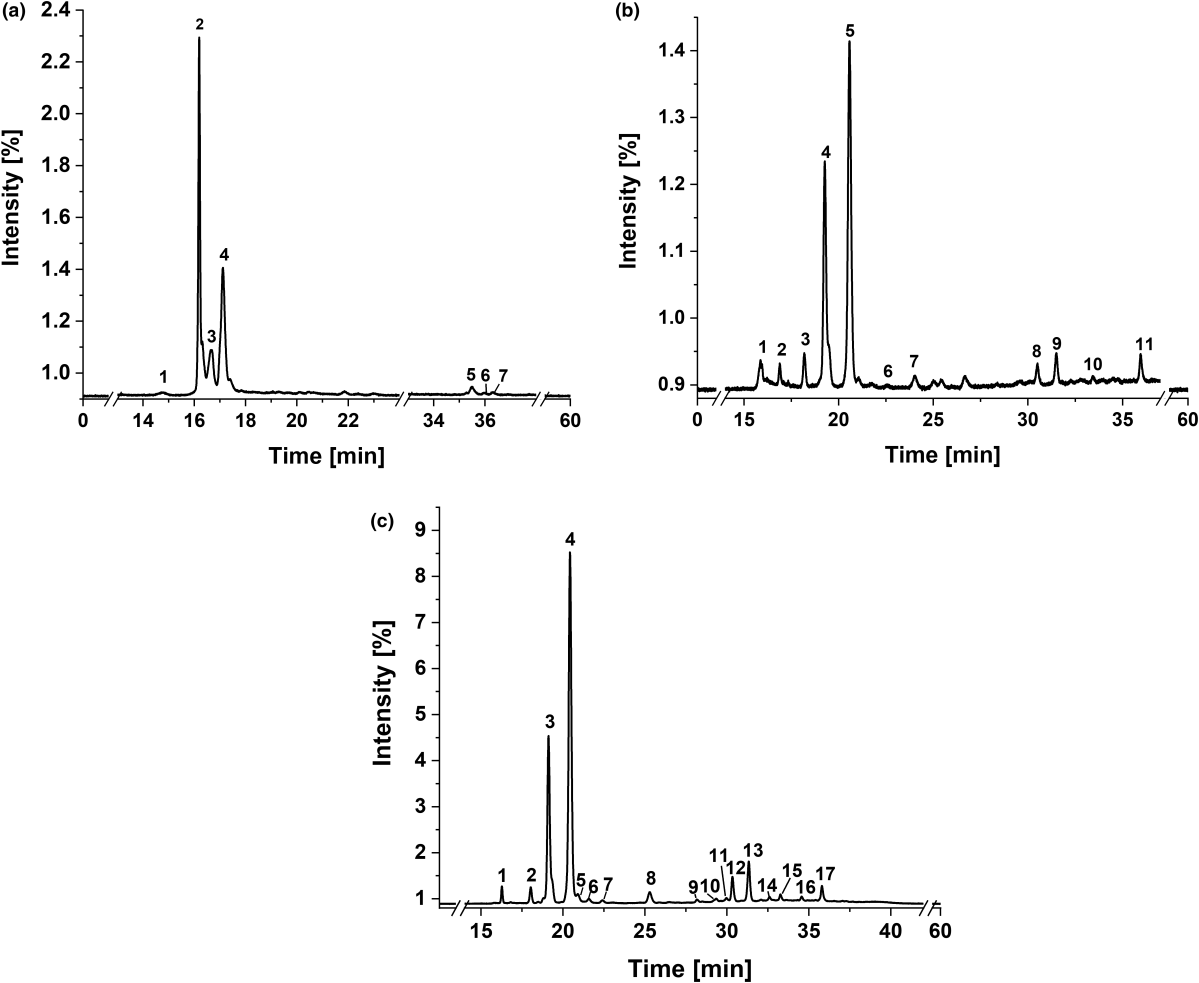
HPLC‐DAD chromatograms (λ = 300 m) of (a) A5 (acetone extract of BSG 2, defatted), (b) HA2 (acetone extract of hydrolysis residue of BSG 2), and (c) HE2 (ethyl acetate extract of alkaline hydrolysis of BSG 2) extract; numbering of signals, which were further analyzed by MS/MS experiments; data presented in Tables 2, 3, 4

Extracts were analyzed by HPLC‐UV‐ESI‐MS/MS using two different systems but the same HPLC method; specific MS parameters are shown in the results (Figure 1). For each extract group (A, HE and HA), a HPLC‐DAD chromatogram recorded at 300 nm is presented in Table 2, 3, 4. In general, chromatograms within one extract group were very similar to each other and independent of the BSG batch recorded.

**TABLE 2:** Identification of compounds in BSG A extracts by HPLC‐ESI‐MS/MS and HPLC‐ESI‐MS (negative and positive mode, *t*_R_: retention time, DP: declustering potential, CE: collision energy; x: compound contained; (x): only precursor ion detected in MS/MS mode; −: compound not detected); fragment ions listed in order of decreasing intensity

Peak	Compound (tentative identification)	*t*_R_ (min)	[M + H]^+^ ^a^ [M–H]^–^ ^b^ (*m/z*)	Fragments (*m/z*)	CE (eV)	DP (V)	Extracts						
A extracts													
							A1	A2	A3	A4	A5	A6	A7
No UV signal	Tryptophan	10.1	205^a^	188, 146, 159, 205, 170, 132	15, 20	100	x	x	–	x	x	–	–
1	Coumaroyl hydroxy‐agmatine	14.7	293^a^	293, 147, 275, 259	20	100	–	x	–	x	x	–	X
2 (overlaid signal)	(+)‐Catechin	16.1	289^b^	123, 109, 137, 97	–40 –50	−100	–	(x)	–	x	x	(x)	(x)
2 (overlaid signal)	Catechin‐dimer/Procyanidin B	16.2	577^b^	125, 245, 203, 151, 137, 109, 289	−50	−100	–	(x)	–	x	–	–	–
2–4	Hordatines see Section 3.2.												
No UV signal	Not identified	21.3	189^a^	125, 170, 97	20	100	–	–	x	–	–	x	–
No UV signal	Not identified	31.5	348^a^	295, 155, 277, 195, 173, 213, 171, 259, 313, 348	20	100	x	x	x	x	x	x	(x)
		34.7					(x)	x	–	x	x	x	–
5	Not identified	34.7	527.6^a^	527, 509, 425.5, 491, 473.5, 455,	20	100	x	x	–	(x)	x	x	(x)
6	Not identified	35.4	367.5^a^	349, 367, 332	20	100	x	x	(x)	x	x	x	X
		36.3					x	x	(x)	x	–	–	X
		37.8					(x)	x	–	–	–	–	(x)
7	Not identified	35.5	365.5^a^	347, 330.5, 365.5, 259, 217	20	100	x	x	(x)	x	(x)	x	X
		36.3					x	x	–	x	x	–	–
		36.8					x	x	–	x	–	–	–
No UV signal	LPE 18:2	37.9	478^a^	478, 337, 460, 306	20	100	x	x	(x)	–	–	x	X
		39.3					x	x	–	x	x	–	(x)
No UV signal	LPE 16:0	40.1	454^a^	313, 454, 436	20	100	x	x	–	x	(x)	–	(x)

^a^ [M + H]^+^.

^b^ [M–H]^−^.

**TABLE 3:** Identification of compounds in BSG HA extracts by HPLC‐ESI‐MS/MS and HPLC‐ESI‐MS (negative and positive mode, *t*_R_: retention time, DP: declustering potential, CE: collision energy; x: compound contained; (x): only precursor ion detected in MS/MS mode; −: compound not detected); fragment ions listed in order of decreasing intensity

Peak	Compound (tentative identification)	*t*_R_ (min)	[M + H]^+^ ^a^ [M–H]^–^ ^b^ (*m/z*)	(*m/z*)	CE (eV)	DP (V)	Extracts		
HA extracts									
							HA1	HA2	HA3
No UV signal	Not identified	9.9	192^a^	83, 101, 139, 59, 99, 157, 175, 69	20	100	x	x	x
1	Not identified	16.1	247^b^	247, 171	−20	100	–	x	–
1 (overlaid signal)	Not identified	16.1	199^a^	139, 121, 199, 97	20	100	x	x	(x)
2	Suberic acid	16.9	173^b^	173, 111, 155, 83	−20	100	–	x	–
3	Not identified	17.3	215^a^	83, 139, 121, 197, 123	20	100	x	x	x
		19.4					x	x	–
No UV signal	Azelaic acid	19.6	187^b^	125, 187, 169, 97	−20	−100	–	x	(x)
4	*p*CA	19.3	163^b^	119, 117, 73, 163	−20	−100	x	x	x
5	FA	20.6	193^b^	134, 178	−20	−100	x	x	x
6–11	DiFA and TriFA see Section 3.3								

^a^ [M + H]^+^.

^b^ [M–H]^−^.

**TABLE 4:** Identification of compounds in BSG HE extracts by HPLC‐ESI‐MS/MS and HPLC‐ESI‐MS (negative ionization mode, *t*_R_: retention time, DP: declustering potential, CE: collision energy; x: compound contained; (x): only precursor ion detected in MS/MS mode; −: compound not detected); fragment ions listed in order of decreasing intensity

Peak	Compound (tentative identification)	*t*_R_ (min)	[M–H]^−^ (*m/z*)	Fragments (*m/z*)	CE (eV)	DP (V)	Extracts					
HE extracts												
							HE1	HE2	HE3	HE4	HE5	HE6
1	CA (probably *cis‐* and *trans‐*isomer)	16.7	179	135, 179, 153	−20	−100	‐	‐	‐	x	x	x
		17.8					x	x	x	x	x	x
1 (overlaid signal)	Suberic acid	16.9	173	173, 111, 155, 83	−20	−100	x	(x)	x	‐	x	x
2	Coumaric acid dimer	19.1	373	163, 327, 283, 119	−20	−100	x	x	x	x	‐	x
3	*p*CA (probably *cis‐* and *trans‐*isomer)	19.3	163	119, 117, 73, 163	−20	−100	x	x	x	x	x	x
		20.4					x	x	x	x	x	x
No UV signal	Azelaic acid	19.6	187	125, 187, 169, 97	−20	−100	x	x	x	x	x	x
4	FA (probably *trans*‐isomer)	20.5	193	134, 178, 149, 193	−20	−100	x	x	x	x	x	x
5	FA (probably *cis*‐isomer)	21	193	134, 178, 149, 193	−20	−100	x	x	x	x	x	x
6–17	DiFAs and TriFAs, see Section 3.3											

Tables 2, 3, 4 present the mass spectral data of compounds identified in the respective extracts by HPLC‐ESI‐MS/MS. Some signals were tentatively identified as hordatines (signal 2–4 in Figure 1a) or DiFAs and oxylipins (signals 6–11 in Figure 1b and signal 6–17 in Figure 1c) but will not be presented within this section since the methods had to be optimized in terms of chromatographic separation. All results regarding those compounds will be discussed in Sections 3.2 and 3.3. Identification was based on comparison of MS (parent ion) and MS/MS (fragment ion) data and the retention time (*t*_*R*_) with those of commercially available standards or published data if no standard was available. Besides polyphenolic compounds, such as hydroxycinnamic acids, hordatines and catechin, tryptophan, phospholipids, and dicarboxylic acids were identified.

Some A extracts (A1, A2, A4, and A5) showed the presence of tryptophan (not visible in chromatogram recorded at 300 nm but at 280 nm, data not shown), an essential amino acid, which was verified using a reference substance. It has already been reported to be present in BSG (Essien & Udotong, 2010), but was not found in extracts from BSG 3 (A3, A6, and A7). This could be due to different storage times since microorganisms can degrade amino acids. Besides hordatines (see Section 3.2), coumaroyl hydroxyagmatine, a monomeric precursor of hordatines, was identified by mean of its fragmentation pattern (von Röpenack et al., 1998). It was not observed in the A1, A3, and A6 extracts, which was not surprising for extract A1 as it was produced from BSG composed of 50% wheat/50% barley grain malt, and hordatines are known to be largely barley‐specific compounds. Extracts from BSG3 did not contain the hordatine precursor except in previously defatted samples (A7). This might be for two reasons. It is possible that the hordatine precursor was concentrated by the defatting process, or the defatting procedure may have caused modifications of the contained components. Catechin derivatives, such as (+)‐catechin and procyanidin B, were detected in some extracts, mainly from BSG 2 (A2, A4, and A5), and were verified by using a reference substance. Catechins have previously been detected in BSG. Procyanidin dimers (*m/z* 577) and catechin (*m/z* 289) were observed recently in BSG extracts from acetone–water liquid–liquid extraction (LLE) (Martín‐García et al., 2019). Both are already reported to be potent inhibitors of α‐glucosidase (Bräunlich et al., 2013; You et al., 2011), indicating that they might contribute to the A extracts inhibitory effects detected in our previous study (Becker et al., 2021). Besides the phenolic compounds, three lysophosphatidylethanolamines (LPEs) (*m/z* 478 and *m/z* 454) were detected by their characteristic fragments of *m/z* 337 and 313 resulting from a loss of *m/z* of 141 (Fang & Barcelona, 1998). Phospholipids accounted for around 9.1% in lipid fractions of BSG, which corresponded to around 11% of the total (Niemi et al., 2012). No single phospholipids have been reported to be present in spent grains so far. Regarding the different BSG batches, no clear correlation was observed, and phospholipids were detected in all batches under study. However, a recently published study demonstrated that phospholipids in milk thistle oil inhibit α‐glucosidase (Harrabi et al., 2021). Therefore, they might also be important regarding the enzyme‐inhibition by the A extracts found in our last study (Becker et al., 2021). Further compounds were detected but could not be identified; most of them eluted in a similar region as the LPEs, indicating relatively high lipophilicity. Additionally, most of them (*m/z* 348, 367, and 365) were observed in up to three isomeric forms. Compounds **6** and **7** only differed by 2 Da, which could indicate that compound **6** was the saturated form of compound **7**. Also, both showed similar fragments corresponding to two times loss of 18 Da, which was probably due to the loss of water. A summary of all identified compounds in A extracts is shown in Table 2.

As expected from the HPLC chromatograms recorded at 300 nm, HA extracts contained different compounds from A extracts (Table 3). The extraction process used to prepare the HA extracts has not been reported in the literature until now. They are best described as LLE (acetone) extracts from alkali pretreated BSG. It is well known that alkaline hydrolysis of BSG releases bound polyphenols, mainly hydroxycinnamic acids, which form part of the cell wall structure as cross linkers (Stefanello et al., 2018). Thus, FA and *p*CA were observed in all three HA extracts and verified by using a reference substance. Besides the monomeric hydroxycinnamic acids, precursor ions of DiFAs and trihydroxy oxylipins were observed (full scan; *m/z* 100–1000; data not shown) and analyzed further by tandem mass spectrometry (see Sections 3.3.1. and 3.4). Moreover, two dicarboxylic acids, i.e., suberic and azelaic acid, were identified by their fragmentation patterns and showed good agreement with the reference substance fragmentation and *t*_R_. Cereals and whole grains are known to be a good source of carboxylic acids (Lohaus et al., 1983) and have been detected in BSG. However, as noted for the A extracts, some compounds remained unidentified.

HE extracts (Table 4) contained similar compounds to those in HA extracts, but the signals for hydroxycinnamic acids were more intense and isomeric forms of *p*CA and FA presenting the same fragmentation pattern were detected. Additionally, two isomers of CA already detected in BSG (Moreira et al., 2013; Verni et al., 2020) were observed in all HE extracts. The isomers for FA, *p*CA, and CA were tentatively assigned as *cis‐* and *trans‐*isomers which can be expected from literature (Callipo et al., 2010) with the *trans*‐isomer being the main naturally occurring one. Moreover, a dimer of coumaric acid was identified by means of its fragmentation pattern (Spínola et al., 2018). As in HA extracts, DiFAs, dehydrotriferulic acids (TriFAs), and oxylipins were indicated in negative full scans (*m/z*: 100–1000 Da; data not shown), and the results are discussed in Sections 3.3 and 3.4.

### 3.2 Characterization of A extracts: Identification of phenolamides and quantification of hordatines

The results of the initial LC–MS (MS) experiments (see Section 3.1.) showed that A extracts differed significantly from HA and HE extracts regarding the main compounds, which were tentatively identified as hordatines according to our previously reported study (Becker et al., 2022). In the frame of this work, 60% acetone was used, as it was described in literature as among the most appropriate solvents for the extraction of polyphenols (Meneses et al., 2013). However, a similar solvent (75% acetone) was also used by Kohyama and Ono to isolate hordatines from barley (Kohyama & Ono, 2013). Enhanced product ion scans were performed to specify each hordatine structure and led to further phenolamides being detected. Also, the total hordatine content as *p*CA‐Eq was determined as lately reported by HPLC‐DAD (Becker et al., 2022).

#### 3.2.1 Identification of phenolamides in extracts

**FIGURE 2:**
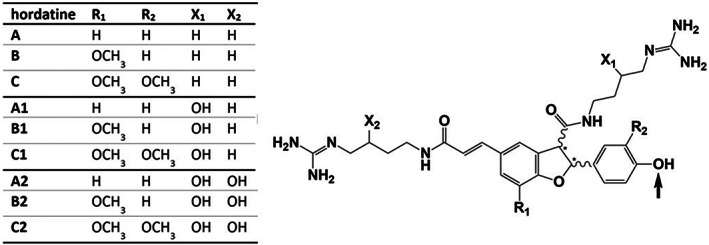
Structures of hordatines found in BSG extracts; all aglycons have been detected, monoglycosidic forms for hordatines A–C and A1–C1, and diglycosidic forms for hordatines A–C; glycosylation (1–3 hexose units) occurs at the hydroxyl group position indicated by the arrow; and sugar moiety: hexose (hex); numbering adapted from published data (Kageyama et al., 2013; Pihlava, 2014)

Phenolamides are an important group of secondary plant metabolites and are also known as hydroxycinnamic acid amides. Their structure comprises at least two components: a phenolic moiety, such as *p*CA, FA, CA, or sinapic acid, and amine part, such as dopamine, tyramine, tryptamine, agmatine, or spermidine, linked to each other by an amide bond. Oligomeric structures and glycosylated forms can also occur and were already reported many years ago for barley. Specific phenolamides, hordatines (Figure 2), which are dimeric forms of hydroxycinnamoyl agmatines, were identified in barley leaves (von Röpenack et al., 1998) in beer where they contribute to astringency (Kageyama et al., 2011; Pihlava et al., 2016) and also in BSG (Becker et al., 2022), which demonstrates their high heat and enzyme stability. Furthermore, some hydroxycinnamoyl putrescines and spermidines were already detected in barley beer (Pihlava, 2014).

Phenolamide structures were clarified by enhanced product ion scans and characteristic fragments according to our previously reported results (Becker et al., 2022) and by means of published data. HPLC‐ESI_pos_‐MS/MS chromatograms and corresponding MS^2^ spectra of some selected hordatines can be found in the Appendix S1: Supplement Material B.

The most characteristic fragment ions for all hordatines are the double‐charged parent ion [M + 2H]^2+^ as well as the fragments resulting from the agmatine moiety for hordatines A, B, and C, and their glycosides (*m/z* 157 (C_5_H_13_N_4_O^+^), 131 (C_5_H_14_N_4_^+^), 114 (C_5_H_14_N_4_^+^ *–*NH_3_), 98 (C_5_H_13_N_4_O^+^ *−* CH_5_N_3_), and 72 (C_5_H_14_N_4_^+^ *−* CH_5_N_3_)) and the hydroxylated agmatine moiety for hordatines A1, B1, C1 and A2, B2, C2, as well as their glycosides (*m/z* 147 (C_5_H_15_N_4_O^+^) and 173 (C_6_H_13_N_4_O_2_^+^)). For glycosidically conjugated hordatines, a neutral loss of 162u resulting from the loss of a hexose unit was also specific.

Besides hordatines, monomeric hydroxycinnamic acid agmatines, such as feruloylagmatine, a precursor of hordatine B and C, were detected. In contrast to the dimeric forms, namely the hordatines, only single‐charged parent ions were found. Characteristic fragments resulted from cleavage of the amide bond and loss of an agmatine moiety to feruloyl (*m/z* 177) and coumaroyl moieties (*m/z* 147).

Similar fragmentation as for feruloylagmatine was also observed for the spermidine conjugates coumaroyl feruloyl spermidine and *bis*‐coumaroyl spermidine. Loss of a polyamine moiety also resulted in *m/z* 177 for the feruloyl and *m/z* 147 for the coumaroyl unit. Based on the enhanced product ion scans, no information about the position of the hydroxycinnamoyl acid on spermidine could be obtained.

As also seen within our last study (Becker et al., 2022), many different isomers (*cis* and *trans,* regio‐isomer, or epimers of the hexose unit) of each hordatine can occur and may be found in the extracts whereby a summary of all phenolamides is shown in Table 5. Regarding the aglycons, the isomers found within this study varied slightly from our previous results. Especially, the amount of double hydroxylated hordatine isomers A2 to C2 was lower within the investigated extracts compared to the isolated fractions in our previously reported study (Becker et al., 2022). This might be due to the much more specified isolation process since the crude isolate which would be comparable to the extracts investigated here (A1*–*A7) was fractionated and thus hordatines were more concentrated in each fraction. Therefore, it might be possible that some isomers are only contained in very small amounts and have not been detected within our extracts. When comparing the different isomers for glycosides, similar differences can be found whereby for some hexosides (e.g., hordatine C and C1 hexoside) more isomers were detected within this study in each extract. Nevertheless, no hexosides of hordatines A2 to C2 were observed in none of the extracts, too. Furthermore, the two processes to extract the hordatines varied and it cannot be excluded that this might also lead to modifications of the original hordatine structure.

**TABLE 5:** Phenolamides (hordatines, hydroxycinnamic acid agmatines, and hydroxycinnamoyl spermidines) found in A1–A7 extracts tentatively identified by double‐charged parent ion and characteristic fragments; x: contained and fragmentation sufficient, (x): insufficient fragmentation due to low concentration, partially only parent ion observed, −: not detected; fragment ions listed in order of decreasing intensity

Phenolamide	*t*_*R*_ (min)	[M + H] ^+^ [M + 2H]^2+^ (*m/z*)	Main fragments (*m/z*)	Extracts						
				A1	A2	A3	A4	A5	A6	A7
*N*‐Feruloylagmatine hexose (hex)	11.8	[M + H] ^+^ 469	469, 307, 177, 145, 117,89	(x)	x	–	x	x	x	x
*N*‐Feruloylagmatine (2 isomers)	14.5	[M + H] ^+^ 307	307, 145, 177, 89, 117	(x)	(x)	–	x	x	–	–
	17.2			x	x	x	x	x	x	x
*N*‐Coumaroylagmatine	15.8	[M + H] ^+^ 277	277, 147, 119, 91	–	x	–	x	x	–	–
*p*‐Coumaroyl feruloyl spermidine	26.2	[M + H] ^+^ 468	468, 147, 177, 204, 117, 234, 322, 452	x	x	–	x	x	(x)	(x)
*bis*‐Coumaroyl spermidine	25.5	[M + H] ^+^ 438	438, 147, 119, 204, 91, 421, 292, 403, 398,	x	x	(x)	x	x	(x)	(x)
Hordatine A (2 isomers)	16.5	276	276, 395, 291, 263, 247, 207, 178, 189, 115, 152, 421, 378, 534,	(x)	x	X	x	x	x	x
	18.5									
				x	x	X	x	x	x	x
Hordatine B (3 isomers)	15.7	291	291, 425, 321, 278, 165, 178, 157, 131, 425, 114, 98, 451	x	x	x	x	x	x	x
	18.2									
	18.6			x	x	x	x	x	x	x
				(x)	x	(x)	x	x	x	x
Hordatine C (2 isomers)	18.7	306	306, 455, 481, 351, 165, 152, 293, 131, 114, 157, 265, 323	x	x	X	x	x	x	x
				x	x	X	x	x	x	x
	19.3									
Hordatine A hexoside (2 isomers)	12.4	357	357, 291, 263, 265, 247, 395, 551, 157, 131, 114	–	x	–	x	x	x	x
				x	x	x	x	x	x	x
	13.9									
Hordatine B hexoside (3 isomers)	11.8	372	372, 321, 293, 295, 291, 278, 425, 235, 157, 131, 114, 98	x	x	(x)	x	x	x	x
	13.7									
	14.5			x	x	x	x	x	x	x
				(x)	x	–	–	x	x	x
Hordatine C hexoside (4 isomers)	12.3	387	387, 351, 455, 307, 157, 114, 131, 325	x	x	–	x	x	x	–
	12.5									
	14.1									
	14.4									
				x	x	–	x	x	x	x
				x	x	x	x	x	x	x
				x	x	x	x	x	x	x
Hordatine A dihexoside (6 isomers)	11.5	438	438, 395, 291, 265, 247, 421, 235, 509, 534, 157, 114, 98, 85	–	–	–	x	x	x	x
	11.8									
	12.3									
				–	x	x	x	x	x	x
	12.8									
	13.4									
	13.7									
				–	–	x	x	x	x	x
				x	x	x	x	x	x	x
				x	x	x	x	x	x	x
				x	x	x	x	x	x	x
Hordatine B dihexoside (4 isomers)	11.7	453	453, 321, 425, 278, 235, 207, 581, 539, 157, 114, 98	x	–	x	x	x	x	x
	12.6									
	13.1									
	13.5									
				x	x	x	x	x	x	x
				x	x	x	x	x	x	x
				x	x	x	x	x	x	x
Hordatine C dihexoside (2 isomers)	12.9	468	468, 455, 351, 594, 325, 306, 481, 611, 157, 756, 98	x	x	(x)	x	x	x	x
	14									
				x	x	x	x	x	x	x
Hordatine A trihexoside (3 isomers)	11.7	519	519, 276, 291, 438,	(x)	x	x	x	x	x	x
	12.3									
				–	(x)	–	(x)	(x)	x	x
	13.2		357, 265, 551, 696, 713, 858, 876	x	x	(x)	x	x	x	x
Hordatine B trihexoside (6 isomers)	11.6	534	534, 291, 453, 581, 265, 247, 743, 726, 905, 564	(x)	x	(x)	x	x	x	x
	12.1									
	12.4									
	13									
	13.4									
	13.8									
				–	(x)	(x)	x	x	(x)	–
				(x)	x	(x)	x	x	x	(x)
				(x)	x	(x)	x	x	(x)	(x)
				(x)	(x)	(x)	(x)	(x)	x	x
				(x)	x	(x)	x	x	(x)	(x)
Hordatine C trihexoside (3 isomers)	12.6	549	549, 306, 611, 773, 756, 351, 455, 935	–	(x)	(x)	–	–	x	x
	13.4									
				(x)	x	(x)	x	x	x	(x)
	13.8			(x)	x	x	x	x	x	x
Hordatine A1 (3 isomers)	14.5	284	284, 393, 189, 178, 115, 291, 421, 438, 411	(x)	–	–	x	x	x	x
	15.4			–	x	x	x	x	x	x
	16.5			x	x	x	x	x	x	x
Hordatine B1 (6 isomers)	12.6	299	299, 278, 165, 425, 321, 176, 189, 130, 293, 283 (only isomers at 12.6 and 14.5 min)	–	–	–	–	–	–	x
				(x)	(x)	(x)	(x)	x	x	x
	13.5									
				x	x	(x)	x	x	x	x
	14.5			–	(x)	–	(x)	x	x	x
				–	x	x	x	x	x	x
	15.3									
				x	x	x	x	x	x	x
	16.2									
	17									
Hordatine C1 (2 isomers)	16.8	314	314, 297, 279, 114, 165, 455, 351, 395	–	x	(x)	–	(x)	(x)	x
				–	x	x	–	(x)	(x)	x
	17.5									
Hordatine A1 hexoside (3 isomers)	11.5	365	365.7, 291, 263, 348, 207, 147, 395	–	x	–	x	(x)	(x)	–
	11.9									
				(x)	x	–	x	x	(x)	–
	12.7									
				(x)	x	x	x	x	x	–
Hordatine B1 hexoside (6 isomers)	10.3	380	380, 321, 293, 295, 277, 178, 165, 425, 564, 147	–	–	–	(x)	x	x	x
	10.9									
				–	(x)	–	(x)	(x)	(x)	(x)
	11.2									
				(x)	–	–	(x)	(x)	(x)	(x)
	11.5									
				x	x	x	x	x	x	x
	12.1									
				–	x	–	–	–	–	–
	12.6									
				x	x	x	x	x	x	x
Hordatine C1 hexoside (5 isomers)	11.4	395	395, 351, 355, 293, 307, 265, 378, 455, 165, 147	–	–	–	–	(x)	(x)	(x)
	11.8									
				–	x	–	–	(x)	(x)	(x)
	12.3			x	x	–	(x)	(x)	(x)	(x)
	13.2			x	x	–	x	x	(x)	(x)
	13.7									
				(x)	x	–	(x)	X	(x)	(x)
Hordatine A2^a^ (1 isomer)	17.7	292	not detectable	(x)	(x)	(x)	(x)	(x)	(x)	(x)
Hordatine B2 (1 isomer)	15	307	307, 278, 293, 165, 178, 130, 441, 321	x	x	x	(x)	x	x	x
Hordatine C2 (1 isomer)	15.5	322	322, 281, 265, 308, 165, 130, 299, 351, 471, 513	(x)	x	(x)	x	(x)	x	(x)

^a^ Isomer at 17.7 min overlapped with hordatine B (*m/z* 291); therefore, there was no clear distinction between the fragments of hordatines B and A2.

Comparing the amount of hordatines present in the extracts and taking into account the much higher measured concentrations for extracts A1*–*A3, it is likely that extracts A4*–*A7 contained considerably more different hordatine structures. This was an expected result for extract A1 since hordatines are known to be barley‐specific compounds and A1 was produced from malt originating from 50% wheat.

Regarding the biosynthetic precursors of hordatines, similar results were already observed in our previously reported study. Both precursors were also found in our hordatine‐rich fractions prepared from BSG (Becker et al., 2022). Also, differences between the BSG batches were observed in the study presented here. *N*‐coumaroylagmatine was only detected in BSG 2 extracts (A2, A4, and A5). Also, *N*‐feruloylagmatine isomers were more dominant in extracts from BSG 2 since the second isomer as well as the glycosylated structure were only both seen in extracts A2, A4, and A5. In addition to agmatine‐containing phenolamides, two hydroxycinnamoyl spermidines were identified. Both were not observed or only in small amounts in extracts from BSG 3 (A3, A6, and A7). Spermidine conjugates have not been identified in BSG so far but were reported to be present in barley (Pihlava, 2014).

#### 3.2.2 Total hordatine content of extracts

**FIGURE 3:**
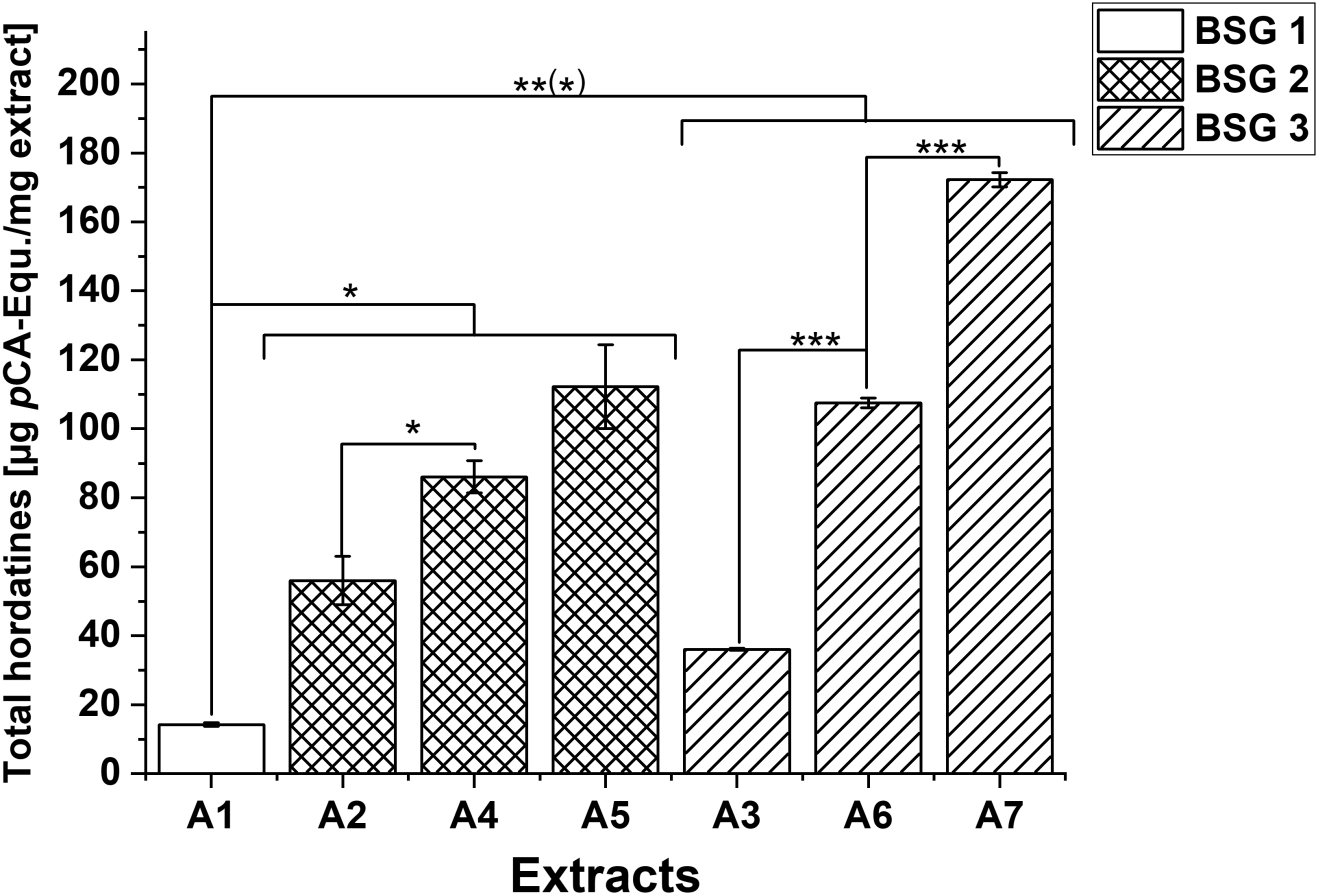
Total hordatine content in A extracts (acetone extraction) expressed as μg *p*‐coumaric acid equivalents (*p*CA‐Eq)/mg extract. Values are expressed as means ± SD of two independent extract solutions each injected three times. Significant differences between different BSG samples and extraction processes were analyzed: *p < .05; **p < .01; ***p < .001

Total hordatine content in A extracts was determined as *p*CA‐Eq as previously described (Becker et al., 2022) and ranged from 14.2 ± 0.5 to 172.2 ± 2.1 μg *p*CA‐Eq/mg extract. The amounts can be considered an estimation of total hordatines in BSG extract due to slight matrix effects and calculation as equivalents. However, significant differences between the extracts were observed (Figure 3). Extract A1, originating from malt with around 50% wheat content, had a significantly lower total hordatine content than all the other A extracts (*p* < .001, .01, and .05), which was expected since hordatines originate from barley. Additionally, differences between extraction processes (see Section 2.2) were observed. Hence, A4 and A6 from process 3 contained significantly more hordatines than the corresponding A2 and A3 extracts from process 1 (*p* < .001, .05). Previous defatting of the raw material (A5 and A7) resulted in higher hordatine content compared to nondefatted samples (A4 and A6), which was significant for A6 and A7 (*p* < .001). To compare our findings with literature data, we calculated the yield‐related hordatine content showing the hordatine content in BSG dw (Appendix S1: Supplement Material B), which should be treated with caution due to the last SPE purification step (Becker et al., 2021). Those BSG‐related values showed similar dependencies between the different BSG batches and extraction processes. Defatted BSG extracts (A5 and A7) had higher total hordatine contents than the corresponding nondefatted extracts (A4 and A6). Extract A1, from 50% wheat share, had the lowest content. The differences between extractions 1 and 2 were not so pronounced.

Quantitative data of hordatines content are relatively limited. The total hordatine content determined as *p*CA‐Eq was only reported for beer by the group of Pihlava et al. (Pihlava et al., 2016) and in our previous study for hordatine‐rich fractions prepared from BSG (Becker et al., 2022). Total hordatines content in beer showed an average value of around 5.6 ± 3.1 mg *p*CA‐Eq/L with maximum value of around 18.7 mg *p*CA‐Eq/L (Pihlava et al., 2016). These values revealed that the relatively high amounts of hordatines present in malt were transferred to wort, and thereafter to the final product beer. However, our previous results demonstrated that hordatines also remained in BSG in high amounts of around 40 μg *p*CA‐Eq /g BS dw. This was confirmed within our study presented here, where total hordatine contents of 242 to 1550 μg *p*CA‐Eq/g BSG dw (Appendix S1: Supplement Material B) were determined. This was even higher than the amounts found in our previously reported study and also than the content observed in beer. However, the main target of the analysis was the identification of bioactive compounds within the BSG extracts. Extracts A2*–*A7 showed strong inhibitory activities towards α‐glucosidase (Becker et al., 2021) and hordatines accounted for a relevant part of the A extracts with up to 17% (extract A7). Our previous reported study demonstrated that the hordatines contribute to the enzyme‐inhibition but the specific active hordatine structure could not be identified. Nevertheless, our findings also indicate a correlation between hordatine content and bioactivity. However, a reference substance is needed for quantification in order to obtain unambiguous conclusions about the hordatine amounts. Also, a validation of the extraction process including repetition and determination of the recovery rates within the SPEs could be performed with a reference substance. Without this evaluation, the calculations of the amount contained in BSG remain merely an estimation.

### 3.3 Characterization of HE and HA extracts: Quantification of hydroxycinnamic acids and structural elucidation of ferulic acid oligomers (FAO)

The results of the initial HPLC–MS(/MS) experiments (see Section 3.1) showed that HA and HE extracts differed significantly from A extracts regarding some of the main compounds tentatively identified as FAOs, which were not observed in A extracts. Since they are mainly part of the cell wall structure they have to be released chemically or enzymatically whereby in the frame of this work alkaline hydrolysis was used. Compared to studies focusing on isolation of FAO (Hernanz et al., 2001; Pedersen et al., 2015), the NaOH concentration was relatively high (4 M), probably leading to higher amounts of monomeric FA than DiFAs. MS/MS experiments were performed to clarify the different structures of FAOs, but only DiFAs could be identified. Furthermore, hydroxycinnamic acids were quantified, whereby the amount of DiFAs was determined as FA‐Eq due to the lack of available reference substances.

#### 3.3.1 Structural elucidation of ferulic acid dehydrodimers (DiFAs) by mass spectrometry (HPLC‐ESI_neg_‐MS/MS)

DiFAs in HA and HE extracts were characterized using tandem mass spectrometry in the negative ionization mode. Characteristic fragment ions and the typical fragment distribution were used to determine the structures of each isomer by means of published data (Callipo et al., 2010). Altogether, a total of 10 DiFAs were identified whereby 2 were only present in low amounts or coeluted with more intense DiFA signals (Appendix S1: Fig. in Supplement Material C). TriFAs, as indicated by the full scans from the first MS experiments (see Section 3.1.), could not be identified by tandem mass spectrometry due to low intensities.

**FIGURE 4:**
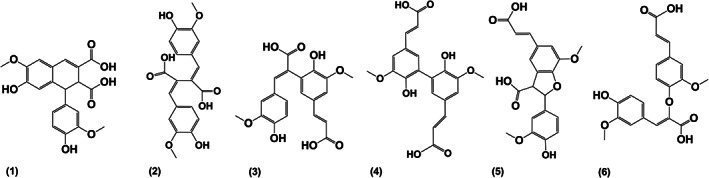
Structures of principal ferulic acid dehydrodimers (DiFAs); trivial names are as follows: (1) 8–8′‐aryltetralin, (2) 8–8′, (3) 8–5′, (4) 5–5′, (5) 8–5′‐cyclic/benzofuran, and (6) 8‐O‐4′ (Callipo et al., 2010; Garcia‐Conesa et al., 1997)

A summary of all DiFAs, their tentative structures according to literature data, and characteristic fragment ions are summarized in Table 6. In general, most isomers were found in HE4*–*HE6 extracts, followed by HE1*–*HE3 and HA1*–*HA3 extracts. All DiFA structures could clearly be distinguished by their specific fragment ions, peak intensities, and comparison with reference data from the literature. The elution order in RP‐HPLC was similar to that published by Callipo et al. who analyzed DiFAs in cereals after liberation from the cell wall structure with slight differences (Callipo et al., 2010): 8–8′‐aryltetralin <8–8′ < 8–5′ < 5–5´ < 8‐O‐4′ form isomers (Figure 4).

**TABLE 6:** DiFAs found in HA1–HA3 and HE1–HE6 extracts tentatively identified; x: contained and fragmentation sufficient, (x): insufficient fragmentation due to low concentration, partially only unspecific fragment ions or signals in TIC detected, −: not detected; characteristic fragments are shown in bold; fragment ions listed in order of decreasing intensity; related HPLC‐MS/MS‐chromatogram can be found in the supplements (Appendix S1: Supplement Material C)

Assumed structure of DiFA	*t*_*R*_ (min)	[M*–*H]^*−*^	Main fragments (*m/z*)	Extracts								
				HA			HE					
				1	2	3	1	2	3	4	5	6
8–8´Aryltetralin DiFA	10.8	385.2	341, 282, 267, **173**, 326, **123**, **217**, 297, 203, **158**, **108**, 311, 239	x	x	x	x	x	x	x	x	x
*trans‐trans*‐8‐8´‐DiFA	11.4	385.2	**159**, 123, **173**, 281, **145**, 267, 341, 108, 91, 297	x	x	x	x	x	x	x	x	x
*trans‐trans*‐8‐5´‐DiFA	13.9	385.2	282, 267, 341, 326, **323**, 311, 308, 385, 239	x	x	x	x	x	x	x	x	x
*trans‐cis‐*8‐8´‐DiFA	14.7	385.2	282, **159**, 123, 267, 281, 297, 326, **145**, **108**, 341	–	–	–	x	x	x	x	(x)	x
*trans‐cis*‐8‐5´‐DiFA	18.4	385.2	341, 267, 282, 326, 311, 297, 249, 239, 323	–	–	–	x	x	x	x	x	x
*trans‐trans‐5‐5*´‐DiFA	23.6	385.2	**385** ^a^, 282, 341, **281**, 326, 267, 370, **265**, 253, 236, 239, 309, 297, 293, 311	x	x	x	x	x	x	x	x	x
8‐O‐4´‐DiFA	26.5	385.2	**193**, **178**, **149**, **134**, 341, 282, 267, 385	x	x	x	x	x	x	x	x	x
8‐O‐4´‐DiFA	28.5	385.2	**193**, **178**, **134**, 311, 282, 326, 385, **149**	(x)	–	(x)	(x)	(x)	x	x	x	x
8‐O‐4´‐DiFA	30.2	385.2	**193**, **178**, **134**, **149**, 385	(x)	–	–	(x)	(x)	x	x	x	x
8‐O‐4´‐DiFA	32.5	385.2	**193**, **178**, **149**, 385	–	–	–	–	–	(x)	x	–	(x)

^a^ Very high stability of parent ion.

For each structure, different diastereoisomers could be observed, e.g., 8–8´‐DiFA and 8‐O‐4´‐DiFA, where two or rather four isomers were detected. In general, *trans‐trans*‐isomers were expected to be the most abundant naturally occurring isomers, followed by *trans‐cis* and *cis‐cis‐*isomers. Isomers in cyclic form may be present in the more stable *anti* or *syn* form.

Many signals appeared to be unspecific as they were detectable for nearly every DiFA structure (*m/z* 385), such as *m/z* 341 (loss of CO_2_), 326 (loss of CO_2_ and CH_3_^.^), 311 (loss of CO_2_ and CH_2_O), 297 (loss of 2x CO_2_), 282 (loss of 2xCO_2_ and CH_3_^.^), and 267 (loss of 2x CO_2_ and CH_2_O). However, each subgroup of DiFAs also showed specific fragmentation patterns, as already reported in detail before (Callipo et al., 2010) and described here in brief. The 8–8′‐aryltetralin isomer (10.8 min; Appendix S1: Fig. Supplement C (1)) showed specific signals at *m/z* 217, 173, 158, 123, and 108, respectively. Linear 8–8′ isomers were found in two forms (eluting at 11.4 and 14.7 min, Appendix S1: Fig. Supplement C (2), (4)). The earlier eluting isomer was more abundant and therefore expected to be the *trans‐trans*‐isomer. Diagnostic signals of *m/z* 173, 159, and 145 were ascribed to cyclic fragments that can only be generated due to the conjugated structure between two aromatic rings. The 8–5´ and 5–5′‐isomers were more difficult to distinguish. One difference was the loss of water of the decarboxylated fragment ion *m/z* 341 for 8–5´‐DiFA (Appendix S1: Fig. Supplement C (3)), leading to a phenolic lactone of *m/z* 323, which was found for the signal at 13.9 min and slightly also for the signal at 18.4 min (Appendix S1: Fig. Supplement C (5)), tentatively assigning them the *trans‐trans*‐ and *cis‐trans‐*8‐5´‐DiFA according to literature data (Callipo et al., 2010). The 5–5´‐DiFA (Appendix S1: Fig. supplement C, (6)) was characterized by a very stable parent ion as well as loss of 60 and 76 Da, giving rise to fragments of *m/z* 281 and 265, which was observed for the signal at 23.6 min. Additionally, it was confirmed using a reference substance (friendly provided by Prof. Dr. Mirko Bunzel, KIT). Since the *anti* configuration is the most stable and the *trans* configuration the naturally occurring one, the isomer found was considered to be the *anti‐trans‐*isomer. All remaining signals (26.5, 28.5, 30.2, and 32.5 min; Appendix S1: Fig. Supplement C (7)–(10)) showed similar signals at *m/z* 193, 178, 149, and 134, corresponding to the typical fragmentation of FA due to the less stable C‐O bond compared to C‐C bonds. Four isomers were observed which were tentatively assigned as *trans‐trans‐, trans‐cis, cis‐trans‐,* and *cis‐cis‐*isomers of 8‐O‐4´‐DiFAs, whereby the main isomer was also verified using a reference substance (Prof. Dr. Bunzel, KIT).

#### 3.3.2 Quantification of hydroxycinnamic acids

**FIGURE 5:**
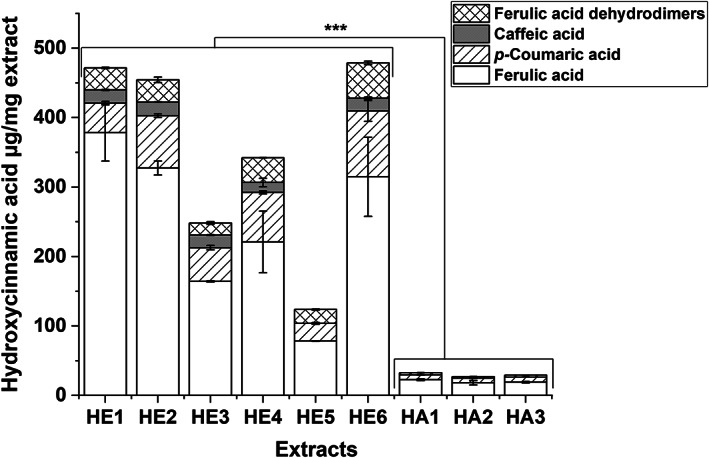
Hydroxycinnamic acid derivatives (CA, pCA, FA, and DiFA) in HA and HE extracts expressed as μg hydroxycinnamic acids/mg extract. Values are expressed as means ± SD of two independent extract solutions each injected two to four times. Significant differences between different BSG samples and different extraction processes were analyzed: ***p < .001. Only significant differences between HE and HA extracts are shown in the Figure

Hydroxycinnamic acid derivatives were quantified in HA and HE extracts by HPLC‐DAD. DiFAs were quantified as FA‐Eq and no CF could be calculated. Thus, the results, already expressed as equivalents, were considered to be an estimation. However, differences between the BSG batches and extraction groups could be evaluated. All extracts contained relatively high amounts of hydroxycinnamic acid derivatives. HA extracts showed significantly lower contents (*p* < .001) than HE extracts for all four groups—CA, *p*CA, FA, and DiFAs—with the exception of CA in HE5, whose content was below the LOQ (Figure 5). Altogether, the hydroxycinnamic acid content accounted to around 3% of the total HA extract and up to 48% of the total HE extract. Regarding our main goal of the study, the identification of active compounds especially the large percentage in the HE extracts is interesting. The HE extracts investigated showed inhibitory activity toward glycogen phosphorylase α (GPα) and α‐glucosidase (Becker et al., 2021). Hydroxycinnamic acids such as FA and DiFAs are reported to be potent inhibitors of different enzymes of the glucose metabolism (Adisakwattana, 2017; Narasimhan et al., 2015; Ye et al., 2022). Thus, their contribution to the enzyme‐inhibition detected in our study (Becker et al., 2021) is likely. The huge difference between the hydroxycinnamic acid contents in HA compared to HE extracts was expected since HA extracts were prepared from the residue of alkaline hydrolysis with the latter serving to produce the HE extracts; i.e., huge amounts of hydroxycinnamic acids were already extracted during the alkaline hydrolysis (HE extracts) whereby only the remaining hydroxycinnamic acids in the residue were extracted with acetone (HA extracts).

For all extracts, the contents could be ranked as follows: FA > *p*CA > DiFA > CA; data presented in Table 7. Similar results for BSG extracts from alkaline hydrolysis have already been reported. Stefanello et al. detected around 103 mg *p*CA/100 g BSG and twice the amount of *trans*‐FA (Stefanello et al., 2018); Alonso‐Riaño et al. (2020) determined FA contents of 1305 μg/g BSG and 538 μg *p*CA/g BSG; and Birsan et al. reported contents of 845 μg FA/g BSG and 387 μg *p*CA/g BSG but only 0.33 μg CA/g BSG (Birsan et al., 2019). However, their extraction processes were similar to the process used for the HE extracts, i.e., purification by LLE using ethyl acetate (Becker et al., 2021). Birsan et al. likewise reported a FA content about twice as high as that of *p*CA in BSG dw. In our study, the FA content was around three‐ to fourfold higher than the *p*CA amount in the HE and HA extracts (around 10‐fold higher for HE1). However, the CA content in HE extracts was much higher than that reported by Birsan et al. All quantitative results reported in the literature used for comparison were provided in mg/g BSG. Therefore, to compare the amounts observed in our study, we calculated the hydroxycinnamic acid derivative amount in BSG dw in relation to extract yield that had previously been published (Becker et al., 2021). It should be mentioned that these calculations only provided an estimation since many purification steps (SPE and LLE) were performed without determination of recovery rates. As the extraction was only performed once, no validation was available. Yield‐related amounts were as follows: for HE extracts: 1251.5 ± 6.9–3283.2 ± 19.8 μg FA/g BSG, 338.7 ± 19.4–970.1 ± 63.1 μg *p*CA/g BSG, n.d. –366.6 ± 15.4 μg CA/g BSG, and 254.5 ± 6.8–507.8 ± 25.1 μg FA‐Eq/ g BSG; for HA extracts: 543.3 ± 93.7–673.6 ± 41.6 μg FA/g BSG, 176.0 ± 8.4–260.4 ± 8.5 μg *p*CA/g BSG, and 65.6 ± 4.2–90.3 ± 8.6 μg FA‐Eq/g BSG. The hydroxycinnamic acid content observed not only in our HE extracts but also in our HA extracts produced from the alkaline hydrolysis residue was much higher or at least in a comparable range than reported values, which may be due to the SPE used for purification and concentration of polyphenols.

**TABLE 7:** Hydroxycinnamic acid contents in HA and HE extracts expressed as μg/mg extract and μg FA‐Eq/ mg extract ± SD

Extract	FA (μg/mg extract) ± SD	*p*CA (μg/mg extract) ± SD	CA (μg/mg extract) ± SD	DiFA (μg FA‐Eq/mg extract) ± SD
HA1	22.55 ± 1.23	7.04 ± 0.34	n.d.	2.92 ± 0.38
HA2	18.11 ± 3.12	6.77 ± 0.80	n.d.	2.19 ± 0.14
HA3	19.08 ± 1.18	7.38 ± 0.24	n.d.	2.56 ± 0.81
HE1	378.57 ± 41.02	42.33 ± 2.43	18.99 ± 0.81	31.82 ± 0.85
HE2	327.40 ± 9.97	75.38 ± 2.76	19.56 ± 0.39	32.23 ± 4.02
HE3	164.16 ± 0.99	48.51 ± 3.16	18.33 ± 0.77	17.13 ± 1.94
HE4	221.00 ± 44.32	71.33 ± 2.10	14.33 ± 6.08	35.50 ± 0.32
HE5	78.22 ± 0.43	25.58 ± 1.41	<LOQ	19.84 ± 0.75
HE6	314.80 ± 56.83	94.85 ± 15.06	18.35 ± 1.55	50.78 ± 2.51

n.d., not detected.

Regarding FAO quantification, Verni et al. recently reported amounts in BSG (Verni et al., 2020). They distinguished between free and bound polyphenols by using LLE with organic solvents as the first extraction step and alkaline hydrolysis as the second step. Hydroxycinnamic acids were not detected in extracts from LLE, except for some dihydrohydroxycinnamic acids. High amounts of hydroxycinnamic acids were observed in extracts after alkaline hydrolysis, i.e., 53 mg/kg FA, 312 mg/kg isoferulic acid, 41 mg/kg *p*CA, 35 mg/kg CA, 568 mg/kg DiFAs, 450 mg/kg tetrameric FA, and 87 mg/kg TriFA. The amounts of monomeric hydroxycinnamic acids determined by Verni et al. were much lower than those detected in our HE and HA extracts, except CA, which was not observed in HA extracts. However, the DiFA content was significantly lower in HA extracts and about half the amount up to the same value in HE extracts. We did not find any trimeric or tetrameric FA in detectable amounts. This might be due to slight differences in the extraction processes. Firstly, 4 M NaOH was used for the preparation of HE and HA extracts, whereas Verni et al. (2020) used 2 N NaOH. A higher concentration of alkali might result in higher hydrolysis rates of the cell wall structure, and therefore higher amounts of monomeric instead of oligomeric structures. Furthermore, about 20 years ago, Hernanz et al. quantified hydroxycinnamic acids in BSG whereby DiFAs were analyzed as single isomers using synthesized reference substances instead of quantification as FA‐Eq (Hernanz et al., 2001). They found 565 to 794 μg/g BSG *p*CA, 1860 to 1948 μg/g BSG FA, 119 to 171 μg/g BSG 8–5´ DiFA open form, 150 to 160 μg/g BSG 5–5´ DiFA, 443 to 526 μg/g BSG 8‐O‐4´‐DiFA, and 181 to 232 μg/g BSG 8–5´‐DiFA benzofuran form, depending on the malt used for brewing. Their determined contents are much higher than observed by Verni et al. as well as our results, which might be due to the higher alkaline concentration and differences in the quantification method used.

Some differences were observed between the different BSG batches. Among the HA extracts, no major differences between the BSG batches were detected. However, this was not the case for HE extracts. Extracts from BSG3 (HE3 and HE5) showed significantly (*p* < .001 and *p* < .01) lower FA contents than all other HE extracts. This also applied to the *p*CA contents (*p* < .001), except HE1, which showed relatively low *p*CA content. The HE6 extract produced from defatted BSG3 displayed the highest amounts of all hydroxycinnamic acid derivatives within extracts HE3*–*HE6, indicating that the defatting process enhanced the extractability of hydroxycinnamic acids. This effect was also observed for the HA extracts. Furthermore, the extraction process of HE1*–*HE3 extracts seemed to be more effective in extracting hydroxycinnamic acids than the method used for HE4*–*HE6 extracts, which were seen in higher amounts for all monomeric hydroxycinnamic acid contents in extracts from process 2. The main difference between the two extraction processes (Table 1) was the order of the two steps. For HE4*–*HE6, the BSG raw material was directly treated by alkaline hydrolysis, whereas for HE1*–*HE3, the BSG was first extracted with 60% acetone and the residue was used for alkaline hydrolysis. A similar study was recently performed by Ideia et al. (Ideia et al., 2020). However, in contrast to our results, they did not find significantly higher FA content for the acetone pretreated samples and concluded that acetone did not effectively release FA from lignocellulosic material (Ideia et al., 2020). In our study, the different content of hydroxycinnamic acids might be due to different volumes of NaOH used for the extraction. For extracts HE4*–*HE6, a ratio of 10 ml NaOH/g BSG was used, whereas nearly three times the amount of 27 ml NaOH/g BSG was used for the preparation of HE1*–*HE3 (Becker et al., 2021). This was to obtain better performance in the follow‐up LLE with ethyl acetate. The influence of NaOH concentration on the extractability of FA and *p*CA has already been investigated. Mussatto et al. demonstrated that reaction time, temperature, and concentration of NaOH influenced the hydroxycinnamic acids content and found the highest values for the maximum conditions of 2% NaOH (% w/v), 90 min, and 120°C (Mussatto et al., 2007).

### 3.4 Identification of oxylipins in BSG extracts

Besides polyphenolic compounds, such as hydroxycinnamic acids and hordatines, hydroxylated fatty acids were detected in all extracts (HA, HE, and A) under study. Different isomers of trihydroxyoctadecenoic (TriHOME) and trihydroxyoctadecanoic acid (TriHODA) were identified by HPLC–MS/MS by means of published MS data (Bhunia et al., 2018; Martin‐Arjol et al., 2010) and partially available reference substances. All of them were linoleic acid‐derived oxylipins. Eight TriHOMEs and two TriHODAs were found in total, with the TriHOME isomer at 28.3 min and the TriHODA isomer at 28.9 min (Appendix S1: Supplement Material D) being the predominant structures in all extracts. It was reported previously that only 4%*–*5% of malt lipids are released into the wort and the majority of lipids remain in the BSG, where probably around 30% of the lipids are oxidized during mashing, which might have generated the oxylipins found in our studies (Anness & Reud, 1985).

An overview of all oxylipins detected in each extract is provided in Table 8. In general, most isomers of TriHOME were found in HE extracts, followed by HA extracts, with the lowest content in A extracts, where only the isomer at 27.5 min was present. Furthermore, TriHODA isomers were not identified in A extracts (except in A1 and A6) but were identified in HA and HE extracts. These findings indicate that most of the oxylipins were (a) released by alkaline hydrolysis, or (b) generated and modified during the extraction process with alkaline solvent. It has already been reported many years ago that TriHOMEs are present in beer (Esterbauer & Schauenstein, 1977) and are produced during the malting and mashing process by lipoxygenase (LOX) reaction, resulting in two isomers: (9, 10, 13)‐ and (9, 12, 13)‐TriHOME, which further represent eight diastereomers and eight enantiomers. However, the enzyme‐catalyzed reaction by barley LOX is highly regio‐ and stereoselective, leading mainly to the formation of (9 S, 12 S, and 13 S)‐TriHOME, which can be found in high amounts in wort and beer. This isomer elutes at 27.5 min, corresponding to the retention time of the isomer found mainly in A extracts. Even if the signal intensities in A extracts were relatively low, the fragment distribution was very similar. This indicates that residues of the characteristic (9S, 12S, 13S)‐TriHOME in beer were extracted by 60% acetone, which was also used for the production of HA extracts. However, (9S, 12S, 13S)‐TriHOME was rarely found in HE extracts, whose main isomer corresponded to the retention time of the standard (9S, 10S, 13S)‐TriHOME at 28.3 min. In general, the TriHOME isomers differ in their fragment pattern, but it was difficult to compare in the frame of our study because most of the isomers coeluted. The large variety of isomers in HE and HA extracts may be due to the alkaline treatment. During the enzyme‐catalyzed reaction to form TriHOMEs, intermediate allylic epoxy alcohols derived from 9‐ and 13‐hydroperoxides are formed (Hamberg, 1991), which can then be hydrolyzed by alkali. This hydrolysis, in contrast to enzymatic cleavage, is not regio‐ or stereoselective, and therefore different isomers can be produced. To distinguish between the TriHOME isomers and to test the above hypothesis, optimization of the method is necessary. Methods to separate 16 different isomers have already been reported (Fuchs et al., 2018). To differentiate the TriHOME isomers characteristic, fragment ions such as *m/z* 127, 129, and 199 can be considered and are specific for structural elements concerning the number of hydroxy groups after the double bond. Martin‐Arjol et al. demonstrated that the fragment ion of *m/z* 127 is typical for 9,10,13‐TriHOME isomers (two hydroxy groups before double bond) whereby *m/z* 129 is mainly observed for 9,12,13‐TriHOME isomers (two hydroxy groups after double bond) (Martin‐Arjol et al., 2010). Due to low‐signal intensities, these fragments were not detected for each isomer within our study but can be referred to as an indication for at least all isomers.

**TABLE 8:** Oxylipins found in BSG extracts tentatively identified; x: contained and fragmentation sufficient, (x): insufficient fragmentation due to low concentration, partially only unspecific fragment ions or signals in TIC detected, −: not detected

Oxylipin	*t*_R_ (min)	[M*–*H]^*−*^	Main fragments (*m/z*)	Extracts															
				A extracts							HE extracts						HA extracts		
				1	2	3	4	5	6	7	1	2	3	4	5	6	1	2	3
TriHOME	27.6	329.5	329, 211, 171, 229, 157, 139, 99	x	x	x	x	x	x	–	–	–	(x)	–	–	x	x	x	–
TriHOME	28.3		329, 211, 229, 171, 139, 99	–	–	–	–	–	–	–	x	x	x	x	x	x	x	x	x
TriHOME	29.2		329, 171, 211, 229, 139, 157, 127, 99	–	–	–	–	–	–	–	x	x	x	x	x	x	x	–	x
TriHOME	32.8		329, 211, 199, 171, 129, 229	–	–	–	–	–	–	–	x	–	x	–	x	x	–	(x)	(x)
TriHOME	34.4		329, 201, 171, 129	–	–	–	–	–	–	–	x	x	x	(x)	x	x	(x)	–	(x)
TriHOME	35.2		329, 201, 171, 139, 293, 271	–	–	–	–	–	–	–	(x)	x	x	–	x	x	(x)	–	(x)
TriHOME	36.7		329, 171, 210, 127, 211	–	–	–	–	–	–	–	(x)	x	x	x	x	x	(x)	–	(x)
TriHOME	39.1		329, 199, 181, 211, 129	–	–	–	–	–	–	–	–	x	x	x	x	x	–	(x)	–
TriHODA	28.9	331.4	331, 313, 157, 187, 201, 127, 171, 295	x	–	–	–	–	–	–	x	x	x	x	x	x	x	x	X
TriHODA	31.2		331, 201.5, 195	–	–	–	–	–	x	–	–	–	–	–	(x)	–	–	–	–

Reference substances			
References	*t*_R_ (min)	[M*–*H]^−^	Fragment ions (*m/z*)
9S, 12S, 13S‐TriHOME	27.55	329	229, 211, 329, 183, 193, 171, 129, 99, 293, 311
9S, 10S, 13S‐TriHOME	28.3	329	171, 329, 139, 157, 127, 99, 201, 293, 311, 211

With regard to our main goal, the identification of the active compounds in our extracts, oxylipins, probably does not contribute to the inhibition of the glucose metabolism enzymes α‐glucosidase and GPα (Becker et al., 2021) since they were found in nearly all extracts including the nonactive HA extracts. However, Nadeem et al. reported an inhibition of α‐glucosidase by extracts containing trihydroxy fatty acids (Nadeem et al., 2020). Therefore, our assumption should be confirmed by quantification and isolation of oxylipins followed by the investigation of pure substances in the enzyme‐inhibition assays.

## 4 CONCLUSION

BSG extracts prepared by SLE with 60% acetone (A1*–*A7), SLE with 60% acetone and alkaline hydrolysis of the respective residue (HA1*–*HA3), or alkaline hydrolysis followed by LLE with ethyl acetate (HE1*–*HE6) from three BSG batches (Becker et al., 2021) were analyzed by HPLC‐ESI‐MS/MS. The main compounds were identified and afterward quantified by HPLC‐DAD in order to draw conclusions on the active compounds in the extracts. In general, extracts A1*–*A7 differed strongly from HA1*–*HA3 and HE1*–*HE6 extracts. Catechin, phenolamides, some phospholipids, and tryptophan were detected in extracts A1*–*A7, whereas hydroxycinnamic acids, such as FA and *p*CA as well as DiFAs, were identified in HA and HE extracts. Furthermore, dicarboxylic acids such as azelaic and suberic acids were observed. Moreover, all extracts contained isomers of TriHOME, which have only been reported before in beer (Esterbauer & Schauenstein, 1977). The oxidized forms of linoleic acid were probably generated during mashing by enzymatic reactions, which are likely to be regio‐ and stereoselective, leading to at least one isomer (Garbe et al., 2005). A extracts only contained one TriHOME, whereas HA and HE extracts contained various isomers, which might be due to the alkaline treatment. For the first time, oxylipins were identified in BSG. Also, the identification of phospholipids has not been reported so far. Both lipidic compounds are not well studied regarding the inhibiting potential toward glucose metabolism enzymes, but there are some recently published studies (Harrabi et al., 2021; Nadeem et al., 2020) indicating that the compounds might contribute to the bioactivity observed in our study (Becker et al., 2021).

Extraction of hydroxycinnamic acids from BSG by alkaline treatment is a well‐established method (Hernanz et al., 2001; Verni et al., 2020). Our results are mostly comparable to published data. However, the content of DiFAs was lower and the amounts of monomeric structures higher in our extracts. This indicates that the usage of more concentrated NaOH results in more extensive hydrolysis of the dimeric structures. Differences between the compositions after the use of different extraction methods were observed. HA extracts (prepared by acetone extraction of the residue after alkaline hydrolysis) had significantly lower amounts of hydroxycinnamic acids than HE extracts (prepared from alkaline hydrolysis followed by ethyl acetate extraction). This was expected since acetone was only used to extract the hydrolysis residue and most of the liberated polyphenols were already extracted by the NaOH solution. Hydroxycinnamic acids are already well‐known inhibitors of α‐glucosidase and GPα (Adisakwattana, 2017; Narasimhan et al., 2015) and recently also DiFAs were reported to be potent inhibitors of α‐glucosidase (Ye et al., 2022). Especially for HE extracts, these compounds may be relevant for the inhibition since they account for up to 48% of the total extract. HA extracts did not inhibit the α‐glucosidase, but GPα (Becker et al., 2021). However, HA extracts did not contain DiFAs and only 3% of the total extract accounted for hydroxycinnamic acids. Thus, our results gave strong evidence of these polyphenols to be relevant for the inhibiting potential of the BSG extracts.

Besides hydrolytic extraction methods, SLE has often been used for extraction of polyphenols from BSG with various extraction solvents (Birsan et al., 2019; Bonifácio‐Lopes et al., 2020; Martín‐García et al., 2019) and many phenolic compounds, such as catechin, syringic, and sinapic acid, vanillin, and proanthocyanidins, have been detected. Additionally, some phenolamides, such as hordatines and spermidine conjugates, were observed for the first time in BSG. Altogether, 27 different phenolamide structures were identified, including 2 hydroxycinnamoyl spermidines, 4 hydroxycinnamoyl agmatines, and 21 hordatines, such as hordatines A, B, and C (Pihlava et al., 2016), hordatines A1, B1, and C1, as well as their glucosides and the aglycones hordatines A2, B2, and C2. Most hordatine structures were found in up to six isomeric forms. Furthermore, extraction process 3 (A4*–*A7) yielded more different hordatine isomers compared to A1*–*A3 (from process 1, Table 1). This was expected for A1 since hordatines are barley specific, and BSG 1, used as the A1 raw material, originated from 50% wheat. Relatively high amounts of up to 172 μg *p*CA‐Eq/mg extract corresponding to 1550 μg *p*CA‐Eq/g BSG dw were found, indicating BSG is a good source of phenolamides. Furthermore, hordatines might contribute to the inhibitory potential of A extracts on α‐glucosidase which was also shown in our recently published study (Becker et al., 2022). However, the specific hordatine structure responsible for enzyme‐inhibition is still not clear. Again, our results demonstrate the need for a reference substance for unambiguous quantitative data and investigation of pure substances in the enzyme‐inhibition assay.

All extracts contained various compounds and notable amounts of different phenolic compounds. Alkaline hydrolysis resulted in hydroxycinnamic acid as the main compound (3% of the total HA extracts; up to 48% of the total HE extracts) and SLE with 60% acetone mainly extracted phenolamides such as hordatines (up to 17% of the total A extracts). Both compound classes were present in relatively high amounts. The quantification of the total hordatine content as well as the content of DiFAs has to be interpreted with caution as the results were only semiquantitative owing to the lack of available reference substances. However, differences in the amounts of hordatines and hydroxycinnamic acids between the different BSG batches and extraction methods could be seen as all extracts were analyzed in the same way. Thus, it was shown that extract A1 produced from BSG with 50% barley had the lowest hordatine content, and extracts from BSG 3 contained less hydroxycinnamic acids than BSG 1 and BSG 2 extracts. Nevertheless, for quantification, the precision can be improved by using reference substances. Methods to synthesize or isolate DiFAs have already been reported (Bunzel et al., 2004, 2008).

Nevertheless, our results provide good evidence of the bioactive components in the extracts investigated (Becker et al., 2021) and enable targeted follow‐up studies to be carried out in the future. Thus, hordatines seem to be relevant for α‐glucosidase inhibition and hydroxycinnamic acids including the DiFAs should be focused on both α‐glucosidase‐ and GPα‐inhibition. Finally, the quantification should be improved and the extraction process validated by the use of reference substances to gain precise information on the amounts of bioactive compounds in BSG.

## FUNDING INFORMATION

This research was funded by the EU‐INTERREG project BIOVAL supported by the European Funds for Regional Development, project no. 018‐4‐09‐021.

## CONFLICT OF INTEREST

The authors declare that they have no known competing financial interests or personal relationships that could have appeared to influence the work reported in this article.

## Supporting information


Appendix S1:

## Data Availability

The data presented in this study are available on request from the corresponding author.

## References

[fsn33178-bib-0001] Aarabi, A. , Honarvar, M. , Mizani, M. , Faghihian, H. , & Gerami, A. (2016). Extraction and purification of ferulic acid as an antioxidant from sugar beet pulp by alkaline hydrolysis. Italian Journal of Food Science, 28(3), 362–375. 10.14674/1120-1770/ijfs.v143

[fsn33178-bib-0002] Adisakwattana, S. (2017). Cinnamic acid and its derivatives: Mechanisms for prevention and management of diabetes and its complications. Nutrients, 9(2), 1–27. 10.3390/nu9020163 PMC5331594 28230764

[fsn33178-bib-0003] Alonso‐Riaño, P. , Sanz Diez, M. T. , Blanco, B. , Beltrán, S. , Trigueros, E. , & Benito‐Román, O. (2020). Water ultrasound‐assisted extraction of polyphenol compounds from brewer's spent grain: Kinetic study, extract characterization, and concentration. Antioxidants (Basel, Switzerland), 9(3), 265. 10.3390/antiox9030265 32210202 PMC7139493

[fsn33178-bib-0004] Anness, B. J. , & Reud, R. J. R. (1985). Lipids in wort. Journal of the Institute of Brewing, 91(5), 313–317. 10.1002/j.2050-0416.1985.tb04349.x

[fsn33178-bib-0005] Arts, M. J. T. J. , Grun, C. , de Jong, R. L. , Voss, H.‐P. , Bast, A. , Mueller, M. J. , & Haenen, G. R. M. M. (2007). Oxidative degradation of lipids during mashing. Journal of Agricultural and Food Chemistry, 55(17), 7010–7014. 10.1021/jf070505+ 17637059

[fsn33178-bib-0006] Aura, A.‐M. , Niemi, P. , Mattila, I. , Niemela, K. , Smeds, A. , Tamminen, T. , & Poutanen, K. (2013). Release of small phenolic compounds from brewer's spent grain and its lignin fractions by human intestinal microbiota in vitro. Journal of Agricultural and Food Chemistry, 61(40), 9744–9753. 10.1021/jf4024195 24028071

[fsn33178-bib-0007] Barth, S. J. (2021). BarthHaas report hops 2020/2021 (1st ed.). BarthHaas GmbH 6 & Co KG.

[fsn33178-bib-0008] Becker, D. , Bakuradze, T. , Hensel, M. , Beller, S. , Yélamos, C. C. , & Richling, E. (2021). Influence of brewer's spent grain compounds on glucose metabolism enzymes. Nutrients, 13(8), 2696. 10.3390/nu13082696 34444856 PMC8399999

[fsn33178-bib-0009] Becker, D. , Permann, S. , Bakuradze, T. , Stegmüller, S. , & Richling, E. (2022). Isolation and characterisation of Hordatine‐rich fractions from Brewer's spent grain and their biological activity on α‐glucosidase and glycogen phosphorylase α. Sustainability, 14(14), 8421. 10.3390/su14148421

[fsn33178-bib-0010] Bhunia, R. K. , Showman, L. J. , Jose, A. , & Nikolau, B. J. (2018). Combined use of cutinase and high‐resolution mass‐spectrometry to query the molecular architecture of cutin. Plant Methods, 14, 117. 10.1186/s13007-018-0384-6 30603042 PMC6306009

[fsn33178-bib-0011] Birsan, R. I. , Wilde, P. , Waldron, K. W. , & Rai, D. K. (2019). Recovery of polyphenols from brewer's spent grains. Antioxidants (Basel, Switzerland), 8, 1–12. 10.3390/antiox8090380 PMC6769810 31500308

[fsn33178-bib-0012] Bonifácio‐Lopes, T. , Teixeira, J. A. , & Pintado, M. (2020). Current extraction techniques towards bioactive compounds from brewer's spent grain ‐ a review. Critical Reviews in Food Science and Nutrition, 60(16), 2730–2741. 10.1080/10408398.2019.1655632 31433199

[fsn33178-bib-0013] Bräunlich, M. , Slimestad, R. , Wangensteen, H. , Brede, C. , Malterud, K. E. , & Barsett, H. (2013). Extracts, anthocyanins and procyanidins from Aronia melanocarpa as radical scavengers and enzyme inhibitors. Nutrients, 5(3), 663–678. 10.3390/nu5030663 23459328 PMC3705312

[fsn33178-bib-0014] Bunzel, M. , Funk, C. , & Steinhart, H. (2004). Semipreparative isolation of dehydrodiferulic and dehydrotriferulic acids as standard substances from maize bran. Journal of Separation Science, 27(13), 1080–1086. 10.1002/jssc.200301703 15495409

[fsn33178-bib-0015] Bunzel, M. , Heuermann, B. , Kim, H. , & Ralph, J. (2008). Peroxidase‐catalyzed oligomerization of ferulic acid esters. Journal of Agricultural and Food Chemistry, 56(21), 10368–10375. 10.1021/jf801825z 18841901

[fsn33178-bib-0016] Callipo, L. , Cavaliere, C. , Fuscoletti, V. , Gubbiotti, R. , Samperi, R. , & Laganà, A. (2010). Phenilpropanoate identification in young wheat plants by liquid chromatography/tandem mass spectrometry: Monomeric and dimeric compounds. Journal of Mass Spectrometry, 45(9), 1026–1040. 10.1002/jms.1800 20690165

[fsn33178-bib-0017] Couallier, V. (2013). Statistical models and methods for reliability and survival analysis. In ISTE. Wiley. 10.1002/9781118826805

[fsn33178-bib-0018] Del Río, J. C. , Prinsen, P. , & Gutiérrez, A. (2013). Chemical composition of lipids in brewer's spent grain: A promising source of valuable phytochemicals. Journal of Cereal Science, 58(2), 248–254. 10.1016/j.jcs.2013.07.001

[fsn33178-bib-0019] Essien, J. P. , & Udotong, I. R. (2010). Amino acid profile of biodegraded brewers spent grains (BSG). Journal of Applied Sciences and Environmental Management, 12(1), 103–106. 10.4314/jasem.v12i1.55582

[fsn33178-bib-0020] Esterbauer, H. , & Schauenstein, E. (1977). Isomere Trihydroxyoctadecensäuren in Bier: Beweise für ihr Vorkommen und ihre quantitative Bestimmung. Zeitschrift für Lebensmittel‐Untersuchung und ‐Forschung, 164, 255–259. 910557 10.1007/BF01147300

[fsn33178-bib-0021] European Commission. Joint Research Centre . (2016). Guidance document on the estimation of LOD and LOQ for measurements in the field of contaminants in feed and food. Publications Office. 10.2787/8931

[fsn33178-bib-0022] Fahrmeir, L. , Heumann, C. , Künstler, R. , Pigeot, I. , & Tutz, G. (2016). Statistik (8th ed.). Springer Berlin Heidelberg. 10.1007/978-3-662-50372-0

[fsn33178-bib-0023] Fang, J. , & Barcelona, M. J. (1998). Structural determination and quantitative analysis of bacterial phospholipids using liquid chromatography/electrospray ionization/mass spectrometry. Journal of Microbiological Methods, 33(1), 23–35. 10.1016/S0167-7012(98)00037-2

[fsn33178-bib-0024] Fuchs, D. , Hamberg, M. , Sköld, C. M. , Wheelock, Å. M. , & Wheelock, C. E. (2018). An LC‐MS/MS workflow to characterize 16 regio‐ and stereoisomeric trihydroxyoctadecenoic acids. Journal of Lipid Research, 59(10), 2025–2033. 10.1194/jlr.D087429 30065010 PMC6168300

[fsn33178-bib-0025] Garbe, L.‐A. , Hübke, H. , & Tressl, R. (2005). Enantioselective formation pathway of a trihydroxy fatty acid during mashing. Journal of the American Society of Brewing Chemists, 63(4), 157–162. 10.1094/ASBCJ-63-0157

[fsn33178-bib-0026] Garcia‐Conesa, M. T. , Plumb, G. W. , Kroon, P. A. , Wallace, G. , & Williamson, G. (1997). Antioxidant properties of ferulic acid dimers. Redox Report, 3(4), 239–244. 10.1080/13510002.1997.11747116 27415026

[fsn33178-bib-0027] Hamberg, M. (1991). Trihydroxyoctadecenoic acids in beer: Qualitative and quantitative analysis. Journal of Agricultural and Food Chemistry, 39(9), 1568–1572. 10.1021/jf00009a006

[fsn33178-bib-0028] Harrabi, S. , Ferchichi, A. , Sakhri, H. , Feki, M. , & Hossaineian, F. (2021). Phospholipid and n‐alkane composition, anti‐α‐glucosidase and anti‐cyclooxygenase activities of milk thistle oil. European Food Research and Technology, 247(6), 1557–1567. 10.1007/s00217-021-03732-y

[fsn33178-bib-0029] Hernanz, D. , Nuñez, V. , Sancho, A. I. , Faulds, C. B. , Williamson, G. [. G.]. , Bartolomé, B. [. B.]. , & Gómez‐Cordovés, C. (2001). Hydroxycinnamic acids and ferulic acid dehydrodimers in barley and processed barley. Journal of Agricultural and Food Chemistry, 49(10), 4884–4888. 10.1021/jf010530u 11600039

[fsn33178-bib-0030] Ideia, P. , Sousa‐Ferreira, I. , & Castilho, P. C. (2020). A novel and simpler alkaline hydrolysis methodology for extraction of ferulic acid from brewer's spent grain and its (partial) purification through adsorption in a synthetic resin. Foods (Basel, Switzerland), 9(5), 1–13. 10.3390/foods9050600 PMC7278616 32397105

[fsn33178-bib-0031] Jay, A. J. , Parker, M. L. , Faulks, R. , Husband, F. , Wilde, P. , Smith, A. C. , Faulds, C. B. , & Waldron, K. W. (2008). A systematic micro‐dissection of brewers' spent grain. Journal of Cereal Science, 47(2), 357–364. 10.1016/j.jcs.2007.05.006

[fsn33178-bib-0032] Kageyama, N. , Inui, T. , Fukami, H. , & Komura, H. (2011). Elucidation of chemical structures of components responsible for beer aftertaste. Journal of the American Society of Brewing Chemists, 69(4), 255–259. 10.1094/ASBCJ-2011-0901-01

[fsn33178-bib-0033] Kageyama, N. , Inui, T. , Fukami, H. , & Komura, H. (2013). Structures in the hordatine family with cis ‐cinnamoyl moieties. Cerevisia, 38(2), 55. 10.1016/j.cervis.2013.09.018

[fsn33178-bib-0034] Kohyama, N. , & Ono, H. (2013). Hordatine a β‐D‐glucopyranoside from ungerminated barley grains. Journal of Agricultural and Food Chemistry, 61(5), 1112–1116. 10.1021/jf304453c 23320742

[fsn33178-bib-0035] Kunze, W. (2010). Technology brewing malting (4th ed.). VLB.

[fsn33178-bib-0036] Lohaus, E. , Bios, I. , & Rudiger, W. (1983). Carboxylic acids in wheat, rye and barley. Zeitschrift Für Naturforschung C, 38(7–8), 524–530. 10.1515/znc-1983-7-805

[fsn33178-bib-0037] Lu, S. , & Gibb, S. W. (2008). Copper removal from wastewater using spent‐grain as biosorbent. Bioresource Technology, 99(6), 1509–1517. 10.1016/j.biortech.2007.04.024 17555956

[fsn33178-bib-0038] Lynch, K. M. , Steffen, E. J. , & Arendt, E. K. (2016). Brewers' spent grain: A review with an emphasis on food and health. Journal of the Institute of Brewing, 122(4), 553–568. 10.1002/jib.363

[fsn33178-bib-0039] Mandalari, G. , Faulds, C. B. , Sancho, A. I. , Saija, A. , Bisignano, G. , LoCurto, R. , & Waldron, K. W. (2005). Fractionation and characterisation of arabinoxylans from brewers' spent grain and wheat bran. Journal of Cereal Science, 42(2), 205–212. 10.1016/j.jcs.2005.03.001

[fsn33178-bib-0040] Martin‐Arjol, I. , Bassas‐Galia, M. , Bermudo, E. , Garcia, F. , & Manresa, A. (2010). Identification of oxylipins with antifungal activity by LC‐MS/MS from the supernatant of pseudomonas 42A2. Chemistry and Physics of Lipids, 163(4–5), 341–346. 10.1016/j.chemphyslip.2010.02.003 20188718

[fsn33178-bib-0041] Martín‐García, B. , Pasini, F. , Verardo, V. , Díaz‐de‐Cerio, E. , Tylewicz, U. , Gómez‐Caravaca, A. M. , & Caboni, M. F. (2019). Optimization of sonotrode ultrasonic‐assisted extraction of proanthocyanidins from brewers' spent grains. Antioxidants (Basel, Switzerland), 8(8), 282. 10.3390/antiox8080282 31390772 PMC6721779

[fsn33178-bib-0042] Meneses, N. G. , Martins, S. , Teixeira, J. A. , & Mussatto, S. I. (2013). Influence of extraction solvents on the recovery of antioxidant phenolic compounds from brewer's spent grains. Separation and Purification Technology, 108, 152–158. 10.1016/j.seppur.2013.02.015

[fsn33178-bib-0043] Merten, D. , Erman, L. , Marabelli, G. P. , Leners, B. , Ney, Y. , Nasim, M. J. , Jacob, C. , Tchoumtchoua, J. , Cajot, S. , & Bohn, T. (2022). Potential health effects of brewers' spent grain as a functional food ingredient assessed by markers of oxidative stress and inflammation following gastro‐intestinal digestion and in a cell model of the small intestine. Food & Function, 13(9), 5327–5342. 10.1039/D1FO03090F 35446320

[fsn33178-bib-0044] Michalkiewicz, A. , Biesaga, M. , & Pyrzynska, K. (2008). Solid‐phase extraction procedure for determination of phenolic acids and some flavonols in honey. Journal of Chromatography. A, 1187(1–2), 18–24. 10.1016/j.chroma.2008.02.001 18282581

[fsn33178-bib-0045] Moreira, M. M. , Morais, S. , Carvalho, D. O. , Barros, A. A. , Delerue‐Matos, C. , & Guido, L. F. (2013). Brewer's spent grain from different types of malt: Evaluation of the antioxidant activity and identification of the major phenolic compounds. Food Research International, 54(1), 382–388. 10.1016/j.foodres.2013.07.023

[fsn33178-bib-0046] Mussatto, S. I. , Dragone, G. , & Roberto, I. C. (2007). Ferulic and p‐coumaric acids extraction by alkaline hydrolysis of brewer's spent grain. Industrial Crops and Products, 25(2), 231–237. 10.1016/j.indcrop.2006.11.001

[fsn33178-bib-0047] Mussatto, S. I. , Fernandes, M. , Rocha, G. J. M. , Orfao, J. J. M. , Teixeira, J. A. , & Roberto, I. C. (2010). Production, characterization and application of activated carbon from brewer's spent grain lignin. Bioresource Technology, 101(7), 2450–2457. 10.1016/j.biortech.2009.11.025 20004569

[fsn33178-bib-0048] Mussatto, S. I. (2014). Brewer's spent grain: A valuable feedstock for industrial applications. Journal of the Science of Food and Agriculture, 94(7), 1264–1275. 10.1002/jsfa.6486 24254316

[fsn33178-bib-0049] Nadeem, M. , Mumtaz, M. W. , Danish, M. , Rashid, U. , Mukhtar, H. , Irfan, A. , Anwar, F. , & Saari, N. (2020). UHPLC‐QTOF‐MS/MS metabolites profiling and antioxidant/antidiabetic attributes of *Cuscuta reflexa* grown on *Casearia tomentosa*: Exploring phytochemicals role via molecular docking. International Journal of Food Properties, 23(1), 918–940. 10.1080/10942912.2020.1764578

[fsn33178-bib-0050] Narasimhan, A. , Chinnaiyan, M. , & Karundevi, B. (2015). Ferulic acid exerts its antidiabetic effect by modulating insulin‐signalling molecules in the liver of high‐fat diet and fructose‐induced type‐2 diabetic adult male rat. Applied Physiology, Nutrition, and Metabolism = Physiologie Appliquee, Nutrition Et Metabolisme, 40(8), 769–781. 10.1139/apnm-2015-0002 26201855

[fsn33178-bib-0051] Niemi, P. , Tamminen, T. , Smeds, A. , Viljanen, K. , Ohra‐aho, T. , Holopainen‐Mantila, U. , Faulds, C. B. , Poutanen, K. , & Buchert, J. (2012). Characterization of lipids and lignans in brewer's spent grain and its enzymatically extracted fraction. Journal of Agricultural and Food Chemistry, 60(39), 9910–9917. 10.1021/jf302684x 22963516

[fsn33178-bib-0052] Olajire, A. A. (2020). The brewing industry and environmental challenges. Journal of Cleaner Production, 256, 102817. 10.1016/j.jclepro.2012.03.003

[fsn33178-bib-0053] Pedersen, M. B. , Bunzel, M. , Schäfer, J. , Knudsen, K. E. B. , Sørensen, J. F. , Yu, S. , & Lærke, H. N. (2015). Ferulic acid dehydrodimer and dehydrotrimer profiles of distiller's dried grains with solubles from different cereal species. Journal of Agricultural and Food Chemistry, 63(7), 2006–2012. 10.1021/jf505150g 25660114

[fsn33178-bib-0054] Pihlava, J.‐M. (2014). Identification of hordatines and other phenolamides in barley (*Hordeum vulgare*) and beer by UPLC‐QTOF‐MS. Journal of Cereal Science, 60(3), 645–652. 10.1016/j.jcs.2014.07.002

[fsn33178-bib-0055] Pihlava, J.‐M. , Kurtelius, T. , & Hurme, T. (2016). Total hordatine content in different types of beers. Journal of the Institute of Brewing, 122(2), 212–217. 10.1002/jib.311

[fsn33178-bib-0056] Sahin, A. W. , Hardiman, K. , Atzler, J. J. , Vogelsang‐O'Dwyer, M. , Valdeperez, D. , Münch, S. , Cattaneo, G. , O'Riordan, P. , & Arendt, E. K. (2021). Rejuvenated brewer's spent grain: The impact of two BSG‐derived ingredients on techno‐functional and nutritional characteristics of fibre‐enriched pasta. Innovative Food Science & Emerging Technologies, 68, 102633. 10.1016/j.ifset.2021.102633

[fsn33178-bib-0057] Santos, M. , Jiménez, J. , Bartolomé, B. , Gómez‐Cordovés, C. , & del Nozal, M. (2003). Variability of brewer's spent grain within a brewery. Food Chemistry, 80(1), 17–21. 10.1016/S0308-8146(02)00229-7

[fsn33178-bib-0058] Spínola, V. , Llorent‐Martínez, E. J. , & Castilho, P. C. (2018). Antioxidant polyphenols of Madeira sorrel (*Rumex maderensis*): How do they survive to in vitro simulated gastrointestinal digestion? Food Chemistry, 259, 105–112. 10.1016/j.foodchem.2018.03.112 29680032

[fsn33178-bib-0059] Stefanello, F. S. , Dos Santos, C. O. , Bochi, V. C. , Fruet, A. P. B. , Soquetta, M. B. , Dörr, A. C. , & Nörnberg, J. L. (2018). Analysis of polyphenols in brewer's spent grain and its comparison with corn silage and cereal brans commonly used for animal nutrition. Food Chemistry, 239(239), 385–401. 10.1016/j.foodchem.2017.06.130 28873583

[fsn33178-bib-0060] Szaja, A. , Montusiewicz, A. , Lebiocka, M. , & Bis, M. (2020). The effect of brewery spent grain application on biogas yields and kinetics in co‐digestion with sewage sludge. PeerJ, 8, e10590. 10.7717/peerj.10590 33391884 PMC7761201

[fsn33178-bib-0061] Verni, M. , Pontonio, E. , Krona, A. , Jacob, S. , Pinto, D. , Rinaldi, F. , Verardo, V. , Díaz‐de‐Cerio, E. , Coda, R. , & Rizzello, C. G. (2020). Bioprocessing of brewers' spent grain enhances its antioxidant activity: Characterization of phenolic compounds and bioactive peptides. Frontiers in Microbiology, 11, 1831. 10.3389/fmicb.2020.01831 32849431 PMC7411387

[fsn33178-bib-0062] von Röpenack, E. , Parr, A. , & Schulze‐Lefert, P. (1998). Structural analyses and dynamics of soluble and cell wall‐bound phenolics in a broad spectrum resistance to the powdery mildew fungus in barley. The Journal of Biological Chemistry, 273(15), 9013–9022. 10.1074/jbc.273.15.9013 9535889

[fsn33178-bib-0063] Wannenmacher, J. , Gastl, M. , & Becker, T. (2018). Phenolic substances in beer: Structural diversity, reactive potential and relevance for brewing process and beer quality. Comprehensive Reviews in Food Science and Food Safety, 17(4), 953–988. 10.1111/1541-4337.12352 33350107

[fsn33178-bib-0064] Wellmitz, J. , & Gluschke, M. (2004). Leitlinie zur Methodenvalidierung (1st ed.). Texte. Umweltbundesamt Retrieved from https://www.umweltbundesamt.de/publikationen/leitlinie‐zur‐methodenvalidierung

[fsn33178-bib-0065] Xiros, C. , Topakas, E. , Katapodis, P. , & Christakopoulos, P. (2008). Hydrolysis and fermentation of brewer's spent grain by Neurospora crassa. Bioresource Technology, 99(13), 5427–5435. 10.1016/j.biortech.2007.11.010 18178432

[fsn33178-bib-0066] Ye, C. , Zhang, R. , Dong, L. , Chi, J. , Huang, F. , Dong, L. , Zhang, M. , & Jia, X. (2022). Α‐glucosidase inhibitors from brown rice bound phenolics extracts (BRBPE): Identification and mechanism. Food Chemistry, 372, 131306. 10.1016/j.foodchem.2021.131306 34638069

[fsn33178-bib-0067] You, Q. , Chen, F. , Wang, X. , Luo, P. G. , & Jiang, Y. (2011). Inhibitory effects of muscadine anthocyanins on α‐glucosidase and pancreatic lipase activities. Journal of Agricultural and Food Chemistry, 59(17), 9506–9511. 10.1021/jf201452v 21797278

