# Supplementary material for: Characterization of brewer's spent grain extracts by tandem mass spectrometry and HPLC‐DAD: Ferulic acid dehydrodimers, phenolamides, and oxylipins
Source: Food Sci Nutr. 2022 Dec 21;11(5):2298–320. doi: 10.1002/fsn3.3178 (PMC10171517; doi:10.1002/fsn3.3178)
Supplement: Supplementary file 1 — Appendix S1: [file FSN3-11-2298-s001.zip › FSN3_3178_Supplement A.docx]

**Supplement A:** Detailed information about instrumentation and MS-parameters

Table 1: Instrumentation used for identification and quantification of compounds in BSG extracts

| **system** | **components** | **analysis** |
| --- | --- | --- |
| HPLC-MS 1 | HPLC Agilent 1100 Series with VWD (variable wavelength detector; Agilent Technologies, Santa Clara, California, USA)  PE Sciex API 2000 triple-quadrupole mass spectrometer (SCIEX, Framingham, Massachusetts, USA). | BSG extract characterisation (section 2.3) |
| HPLC-MS 2 | HPLC Agilent 1200 Series  (Agilent Technologies, Santa Clara, California, USA)  Sciex API 3200 triple-quadrupole mass spectrometer (SCIEX, Framingham, Massachusetts, USA) | BSG extract characterisation (section 2.3)  structural elucidation of DiFA and identification of oxylipins  (section 2.5) |
| HPLC-MS 3 | HPLC Agilent 1290 Infinity  (Agilent Technologies, Santa Clara, California, USA)  QTRAP 5500 mass spectrometer  (SCIEX, Framingham, Massachusetts, USA) | identification of phenolamides (section 2.4) |
| HPLC-DAD | HPLC Agilent 1200 Series with diode array-detector (DAD; Agilent Technologies, Santa Clara, California, USA) | quantification of total hordatine content (section 2.6) and hydroxycinnamic acids  (section 2.7) |

Table 2: MS-parameters used for the different analysis methods

| **analysis method** | **MS-parameters** |
| --- | --- |
| ESI_pos_-MS(/MS)-analysis of BSG extracts (section 2.3) | ion spray voltage 4700 V  CUR 10*–*20 psi  nebuliser gas 30*–*40 psi  CAD gas 2 psi  heater gas 30*–*40 psi  T 450 °C  declustering potential (DP) 100 V  focusing potential (FP) 340 V  entrance potential (EP) 8*–*10.5 V  collision cell entrance potential (CEP) 17.5*–*32 V  cell exit potential (CXP) 50 V  collision energy (CE) 15*–*35 eV |
| ESI_neg_-MS(MS)-analysis of BSG extracts (section 2.3) | ion spray voltage −4500 V  CUR 10–20 psi  nebuliser gas 30–40 psi  CAD gas 2–6 psi  heater gas 30-40 psi  T 450 °C  DP −26 to −100 V  FP −160 to −340 V  EP −8 to −11.5 V  CEP −12 to −38 eV  CXP −2 to −15 V  CE −20 to −50 V |
| ESI_pos_-MS(MS)-analysis of phenolamides  (section 2.4) | ion spray voltage 4700 V  CUR 20 psi  nebuliser gas 30 psi  CAD gas −3 psi  heater gas 30 psi  temperature 450° C  DP 100 V  collision energy spread (CES) 30  EP 8 V;  CEP 40 V  CE 30*–*40 eV  LIT fill time 1 msec, dynamic  Q3 entry barrier 8 V  exit lens voltage (EXB) −158.88 to −133.54 V  auxiliary AC 0.14–0.91 V  scan rate 1000 Da/s |
| ESI_neg_-MS(MS)-analysis of DiFAs and oxylipins (section 2.5) | ion spray voltage −4500 V  CUR 20 psi  nebuliser gas 30 psi  CAD gas 5*–*6 psi  heater gas 30 psi  T 450° C  DP −45 to −100 V  EP −10 to −10.5 V  CEP −17 to −31.8 V  CXP −2 to −4 V  CE −20 to −35 eV |
