# Supplementary material for: Characterization of brewer's spent grain extracts by tandem mass spectrometry and HPLC‐DAD: Ferulic acid dehydrodimers, phenolamides, and oxylipins
Source: Food Sci Nutr. 2022 Dec 21;11(5):2298–320. doi: 10.1002/fsn3.3178 (PMC10171517; doi:10.1002/fsn3.3178)
Supplement: Supplementary file 1 — Appendix S1: [file FSN3-11-2298-s001.zip › FSN3_3178_Supplements B.docx]

**Supplements B:** HPLC-ESI_pos_-MS/MS chromatograms of different hordatines and quantitative results of HPLC-DAD analysis


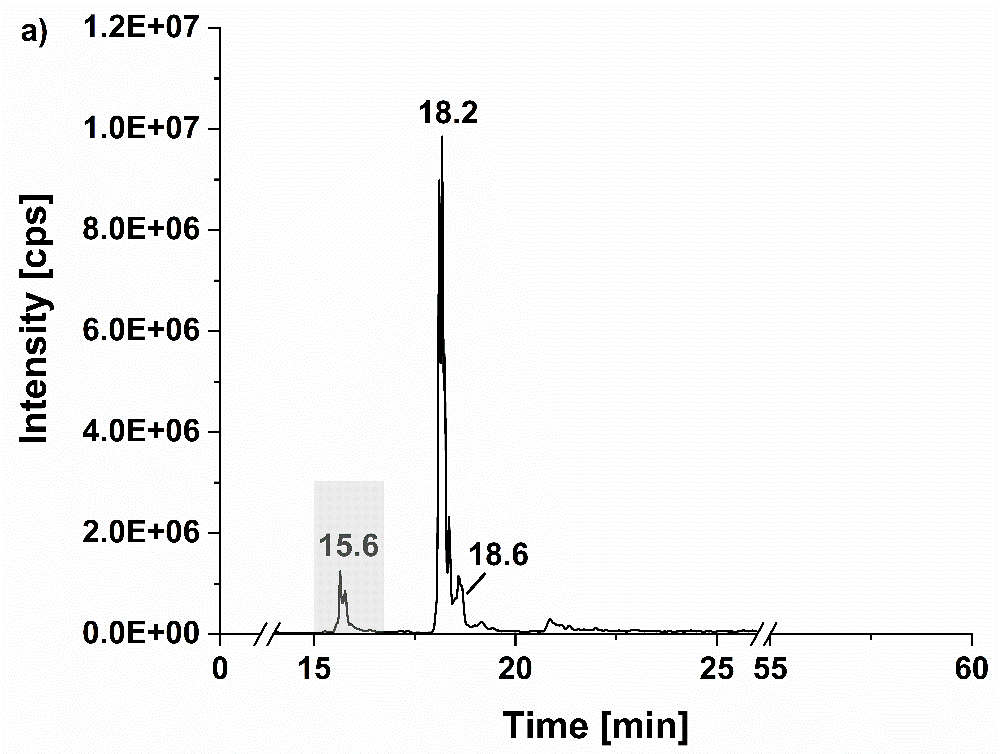

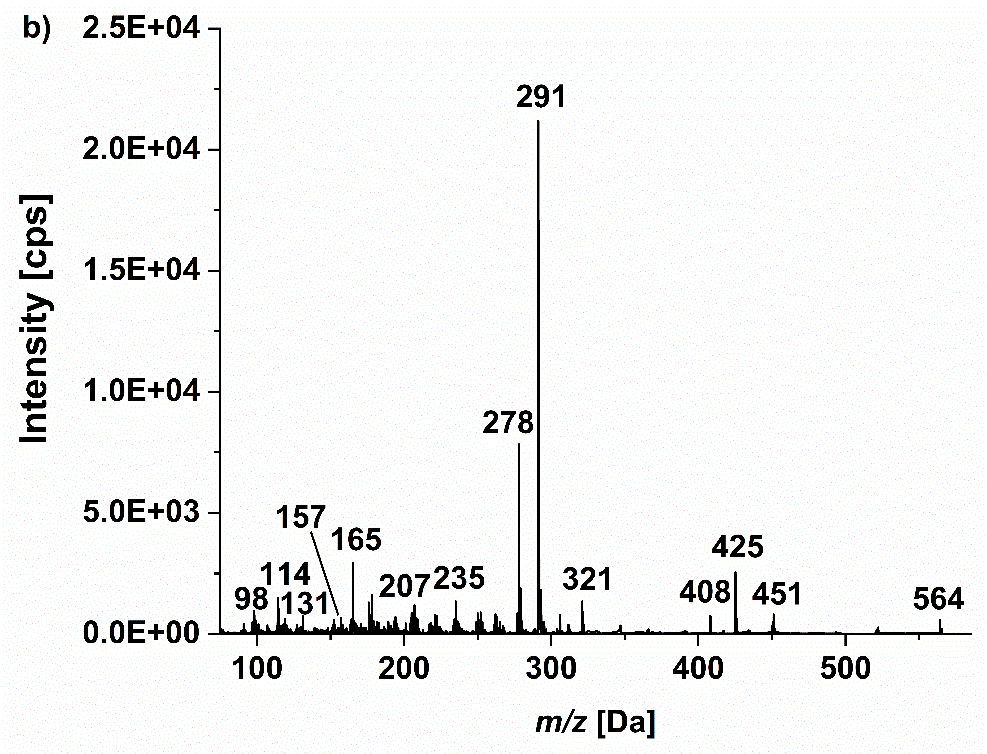


Figure 1: HPLC-ESI_pos_-MS/MS chromatogram of hordatine B (m/z 291, a) and corresponding MS^2^ spectrum of signal at 15.6 min (b) in extract A7 (acetone extract from BSG 3, defatted); CE: 40 eV, CES: 30, DP 100 V.


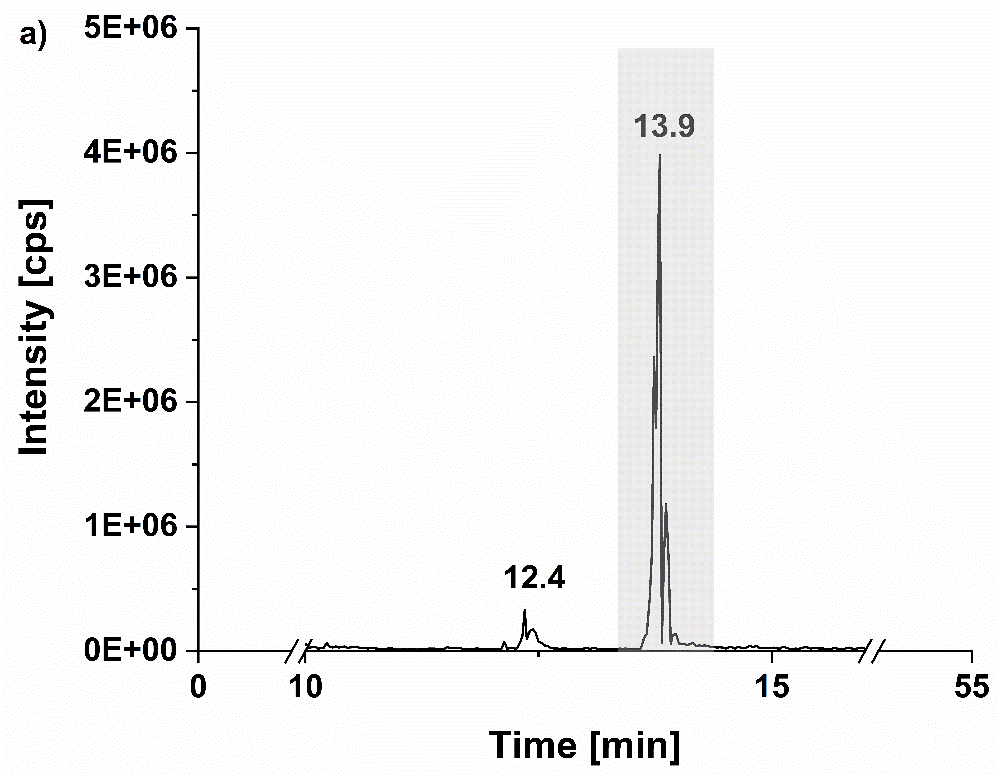

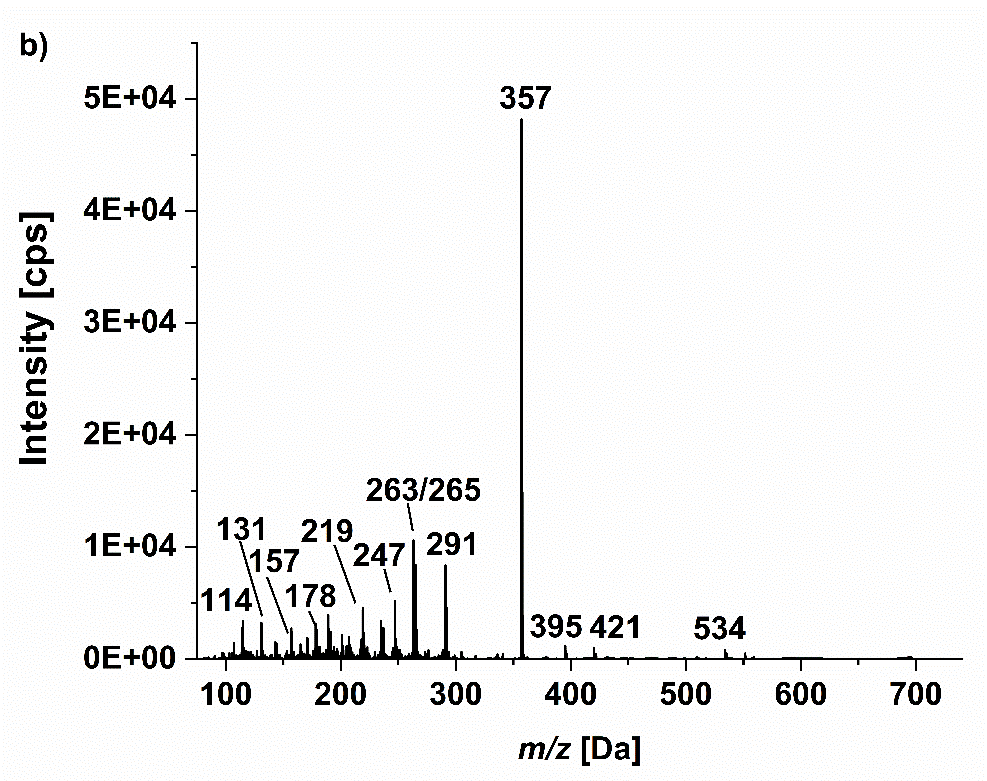


Figure 2: HPLC-ESI_pos_-MS/MS chromatogram of hordatine A hexoside (m/z 357, a) and corresponding MS^2^ spectrum of signal at 13.9 min (b) in extract A7 (acetone extract from BSG 3, defatted); CE 40 eV, CES 30, DP 100 V.


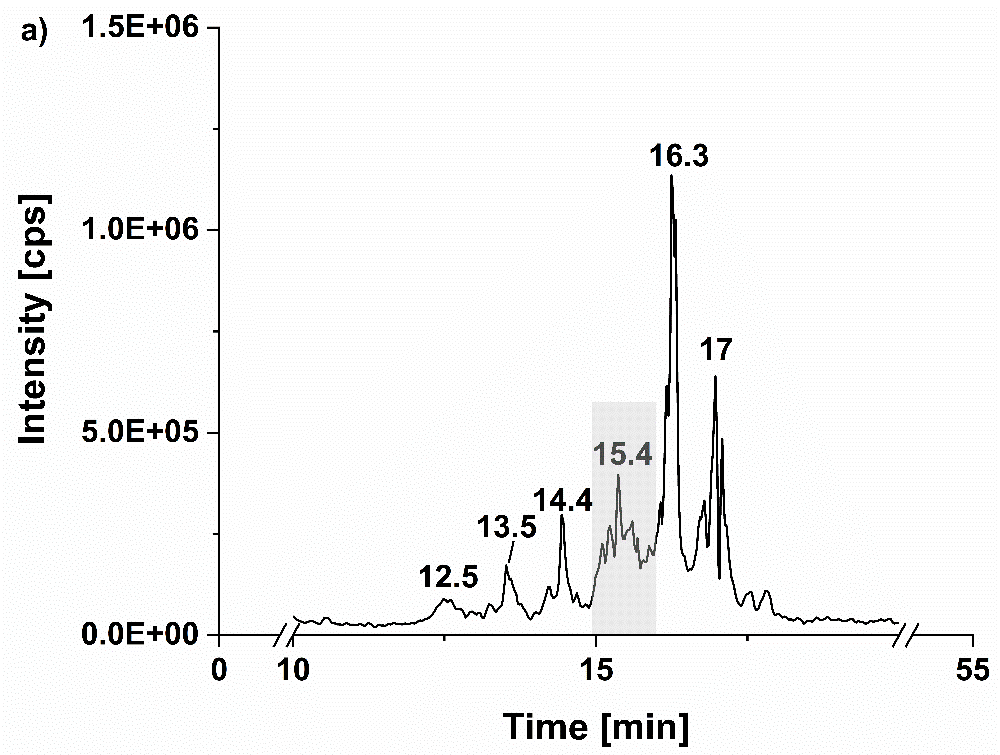

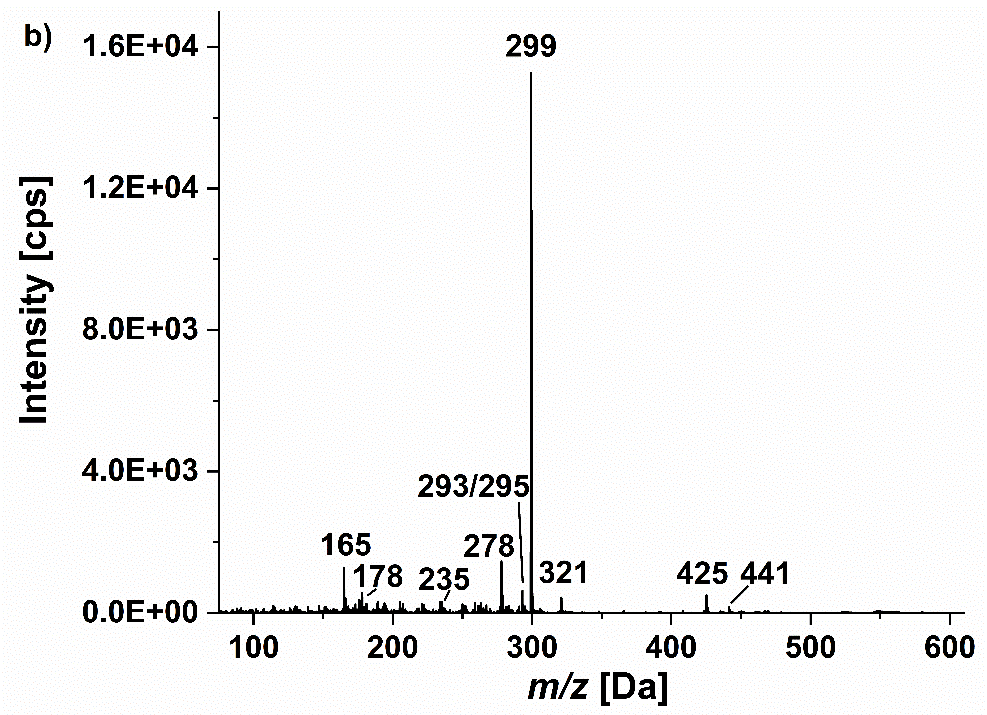


Figure 3: HPLC-ESI_pos_-MS/MS chromatogram of hordatine B1 (m/z 299, a) and corresponding MS^2^ spectrum of signal at 15.4 min (b) in extract A7 (acetone extract from BSG 3, defatted); CE 40 eV, CES 30, DP 100 V.


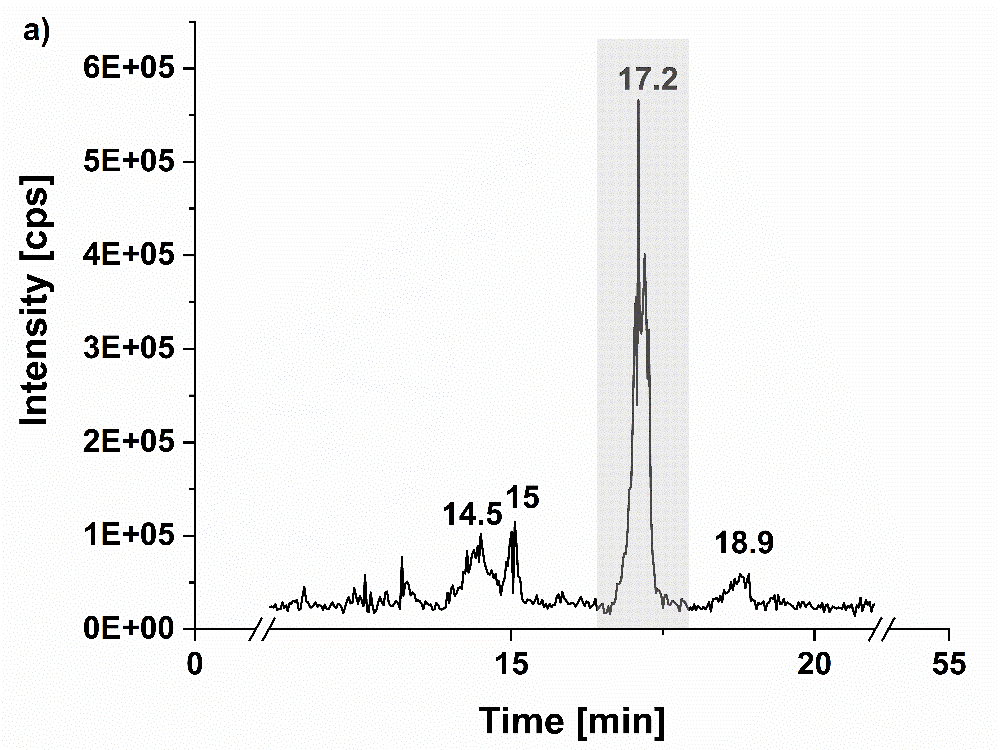

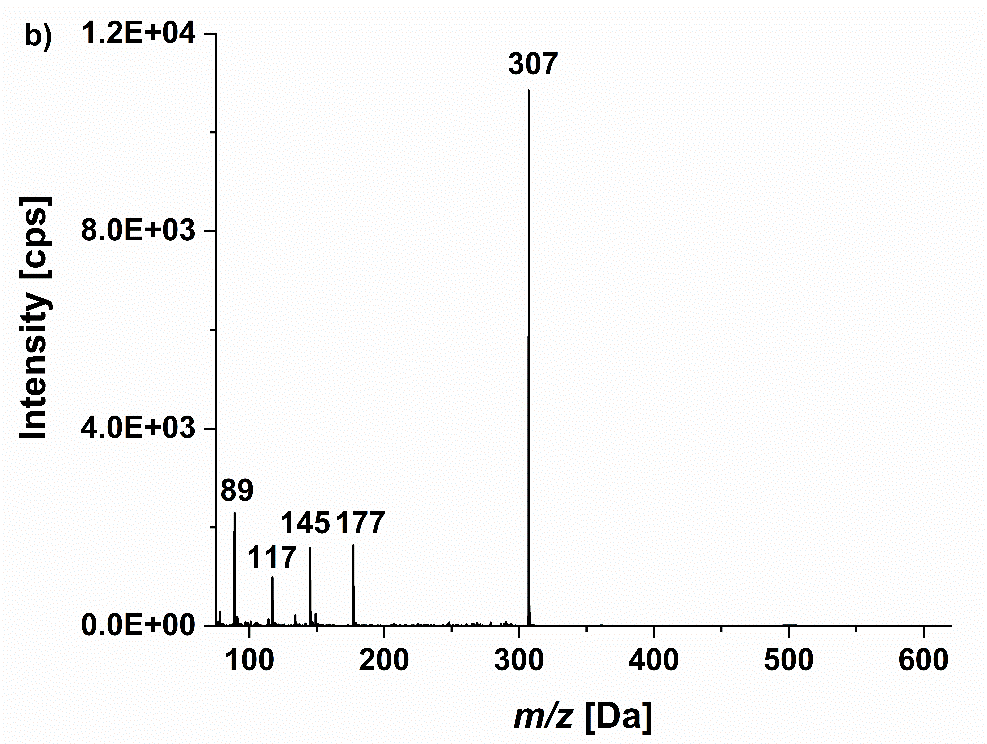


Figure 4: HPLC-ESI_pos_-MS/MS chromatogram of feruloylagmatine (m/z 307, a) and corresponding MS^2^ spectrum of signal at 17.2 min (b) in extract A4 (acetone extract from BSG 2); CE 40 eV, CES 30, DP 100 V.


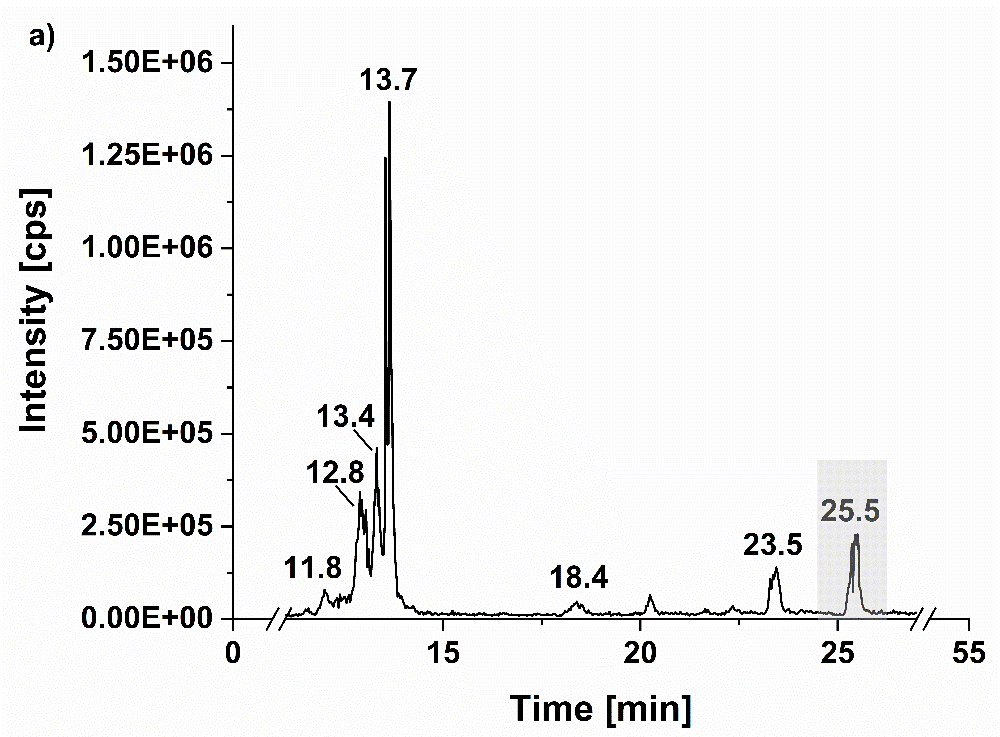

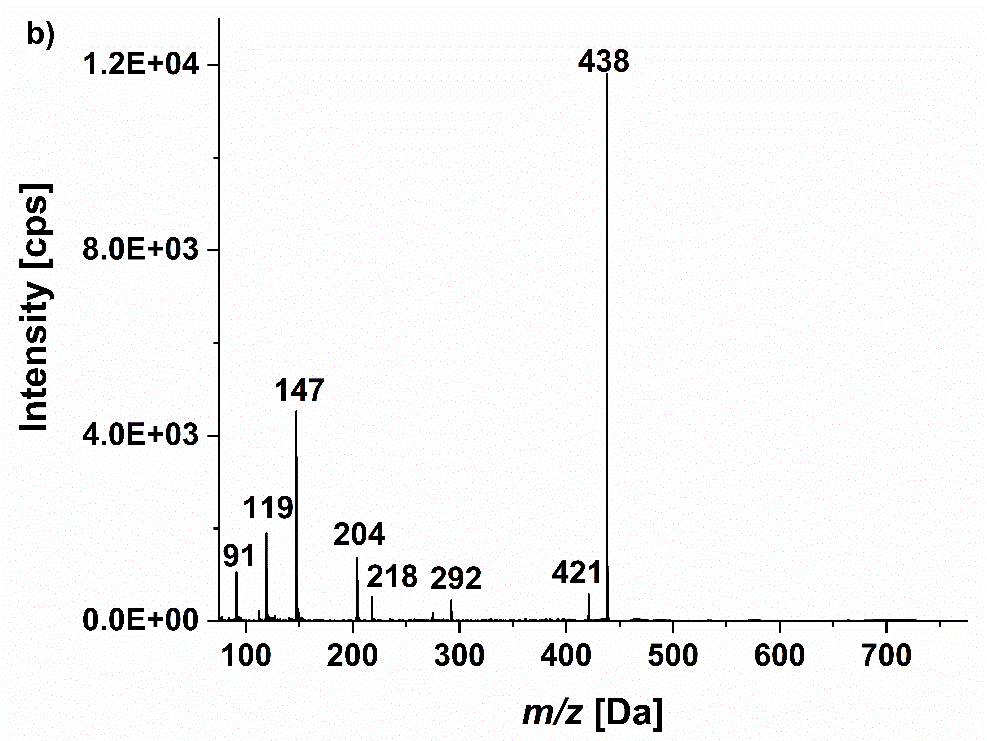


Figure 5: HPLC-ESI_pos_-MS/MS chromatogram of bis-coumaroylspermidine (m/z 438, a) and corresponding MS^2^ spectrum of signal at 25.5 min (b) in extract A2 (acetone extract from BSG 2); CE 40 eV, CES 30, DP 100 V.

Table 1: Total hordatine content in A extracts expressed as µg pCA-Eq/ mg ± SD or yield related in BSG dw as µg pCA-Eq/g BSG dw ± SD.

| Extract | Hordatines  [µg *p*CA-Eq/mg extract] ± SD | | Hordatines (yield related)  [µg *p*CA-Eq/g BSG] ± SD | |
| --- | --- | --- | --- | --- |
| A1 | 14.23 | 0.52 | 242.02 | 8.82 |
| A2 | 55.95 | 7.03 | 671.41 | 84.32 |
| A3 | 36.06 | 0.28 | 829.44 | 6.41 |
| A4 | 86.07 | 4.67 | 688.56 | 37.32 |
| A5 | 112.23 | 12.13 | 897.87 | 97.00 |
| A6 | 107.51 | 1.43 | 967.62 | 12.85 |
| A7 | 172.22 | 2.09 | 1550.06 | 18.80 |
