# Supplementary material for: Characterization of brewer's spent grain extracts by tandem mass spectrometry and HPLC‐DAD: Ferulic acid dehydrodimers, phenolamides, and oxylipins
Source: Food Sci Nutr. 2022 Dec 21;11(5):2298–320. doi: 10.1002/fsn3.3178 (PMC10171517; doi:10.1002/fsn3.3178)
Supplement: Supplementary file 1 — Appendix S1: [file FSN3-11-2298-s001.zip › FSN3_3178_Supplements C.docx]

**Supplements C:** HPLC-ESI_neg_-MS/MS chromatograms of extract HE4





Figure 6: HPLC-ESI_neg_-MS/MS chromatogram of extract HE4 (ethyl acetate extract of alkaline hydrolysis of BSG 2), m/z 385 extracted; CE: −30 eV, DP −45 V.
