# Supplementary material for: Characterization of brewer's spent grain extracts by tandem mass spectrometry and HPLC‐DAD: Ferulic acid dehydrodimers, phenolamides, and oxylipins
Source: Food Sci Nutr. 2022 Dec 21;11(5):2298–320. doi: 10.1002/fsn3.3178 (PMC10171517; doi:10.1002/fsn3.3178)
Supplement: Supplementary file 1 — Appendix S1: [file FSN3-11-2298-s001.zip › FSN3_3178_Supplements D.docx]

**Supplements D:** HPLC-ESI_neg_-MS/MS chromatograms of oxylipins


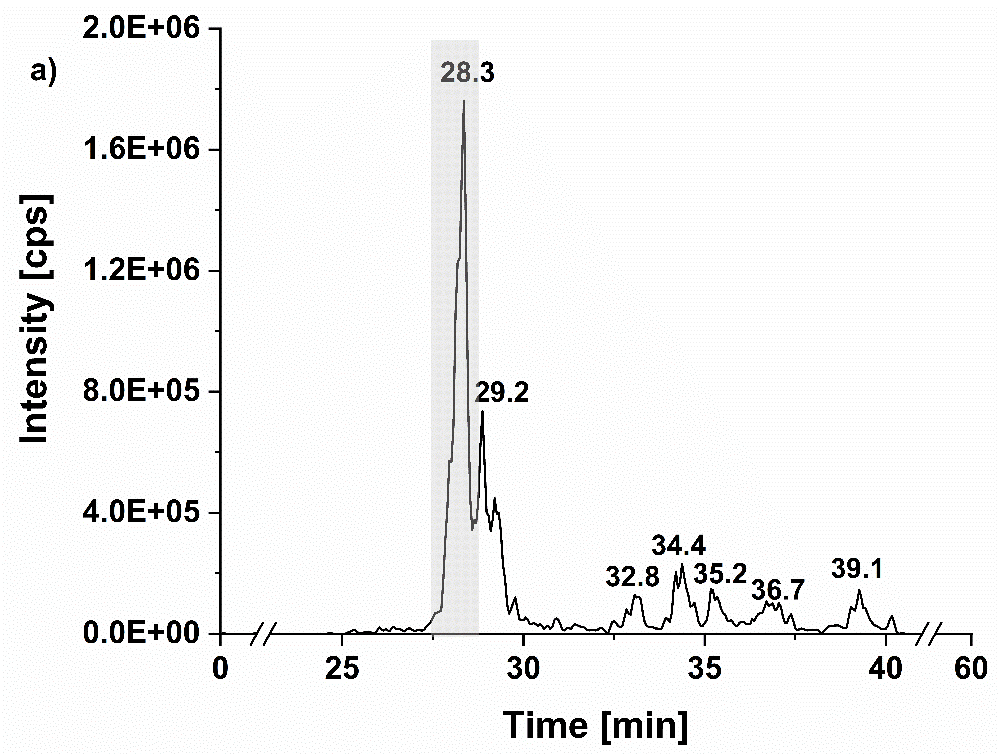

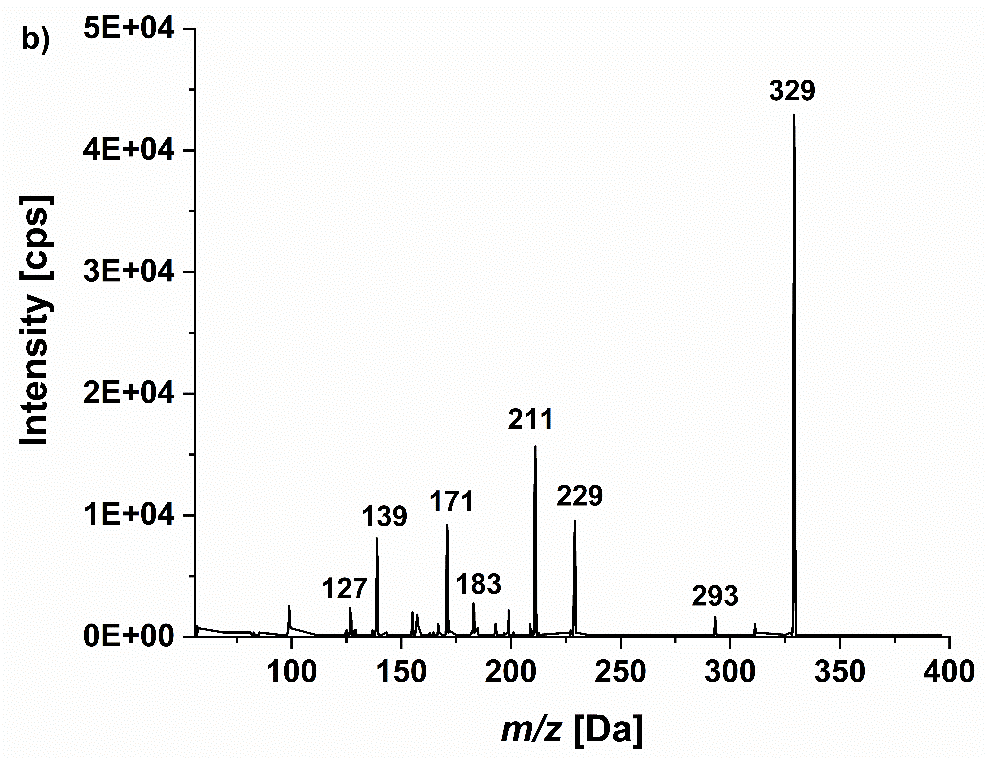


Figure 7: HPLC-ESI_neg_-MS/MS chromatogram of TriHOME isomers (m/z 329, a) and corresponding MS^2^ spectrum of signal at 28.3 min (b) in extract HE5; CE −30 eV, DP −100 V.


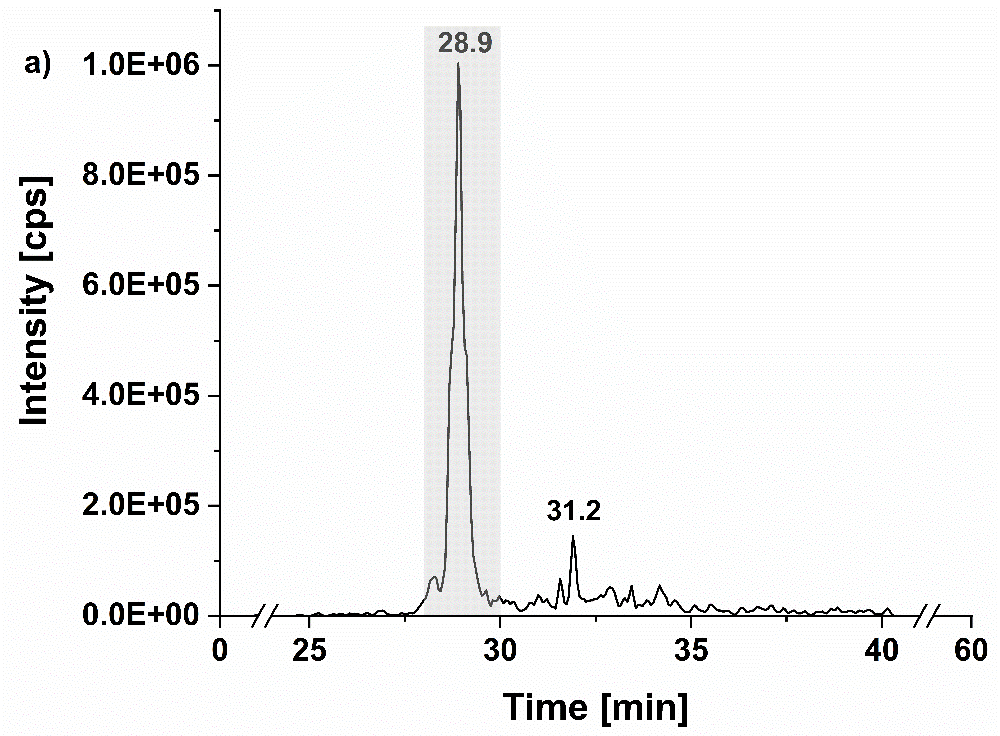

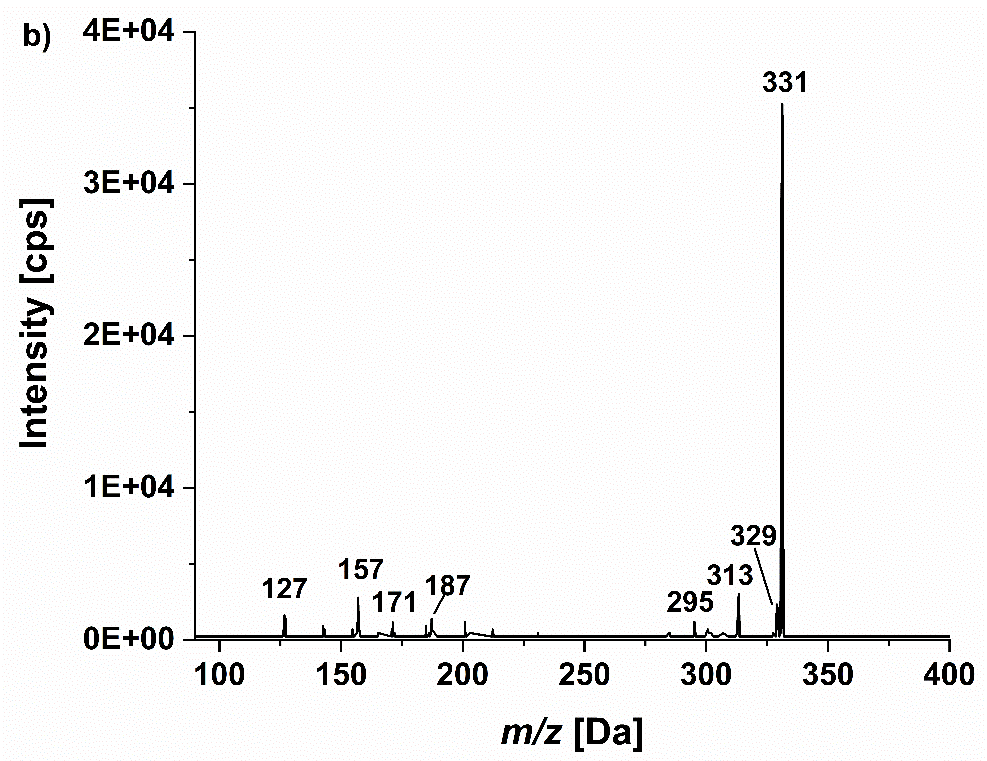


Figure 8: HPLC-ESI_neg_-MS/MS chromatogram of TriHODA isomers (m/z 331, a) and corresponding MS^2^ spectrum of signal at 28.9 min (b) in extract HE5; CE −30 eV, DP −100 V.
